# Discrete analysis of camelid variable domains: sequences, structures, and in-silico structure prediction

**DOI:** 10.7717/peerj.8408

**Published:** 2020-03-06

**Authors:** Akhila Melarkode Vattekatte, Nicolas Ken Shinada, Tarun J. Narwani, Floriane Noël, Olivier Bertrand, Jean-Philippe Meyniel, Alain Malpertuy, Jean-Christophe Gelly, Frédéric Cadet, Alexandre G. de Brevern

**Affiliations:** 1 Biologie Intégrée du Globule Rouge UMR_S1134, Inserm, Univ. Paris, Univ. de la Réunion, Univ. des Antilles, Paris, France; 2 Laboratoire d’Excellence GR-Ex, Paris, France; 3 Faculté des Sciences et Technologies, Saint Denis, La Réunion, France; 4 Institut National de la Transfusion Sanguine (INTS), Paris, France; 5 Discngine SAS, Paris, France; 6 PSL Research University, INSERM, UMR 932, Institut Curie, Paris, France; 7 Université Paris Sud, Université Paris-Saclay, Orsay, France; 8 ISoft, Saint-Aubin, France; 9 Atragene, Ivry-sur-Seine, France; 10 IBL, Paris, France; 11 Peaccel, Protein Engineering Accelerator, Paris, France

**Keywords:** Secondary structure, Nanobodies, Complementarity determining regions, Structural alphabet, Frameworks, Sequence structure relationship, Antibodies

## Abstract

Antigen binding by antibodies requires precise orientation of the complementarity- determining region (CDR) loops in the variable domain to establish the correct contact surface. Members of the family Camelidae have a modified form of immunoglobulin gamma (IgG) with only heavy chains, called Heavy Chain only Antibodies (HCAb). Antigen binding in HCAbs is mediated by only three CDR loops from the single variable domain (V_H_H) at the N-terminus of each heavy chain. This feature of the V_H_H, along with their other important features, e.g., easy expression, small size, thermo-stability and hydrophilicity, made them promising candidates for therapeutics and diagnostics. Thus, to design better V_H_H domains, it is important to thoroughly understand their sequence and structure characteristics and relationship. In this study, sequence characteristics of V_H_H domains have been analysed in depth, along with their structural features using innovative approaches, namely a structural alphabet. An elaborate summary of various studies proposing structural models of V_H_H domains showed diversity in the algorithms used. Finally, a case study to elucidate the differences in structural models from single and multiple templates is presented. In this case study, along with the above-mentioned aspects of V_H_H, an exciting view of various factors in structure prediction of V_H_H, like template framework selection, is also discussed.

## Introduction

**Figure 1:**
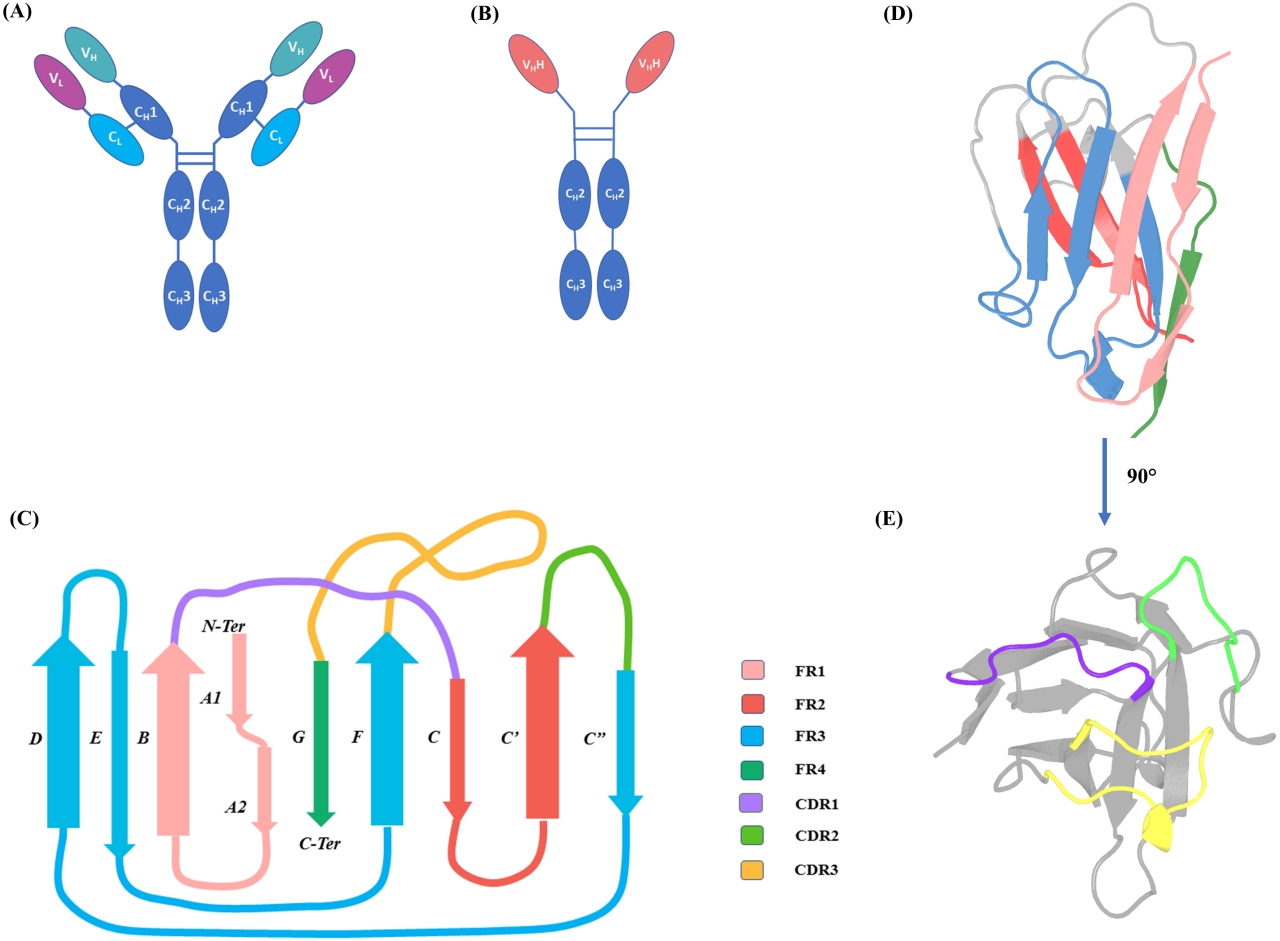
IgG and HCAb. (A) IgG schematic representation with heavy chain (domains V_H_, C_H_1, C_H_2 and C_H_3) and light chain (domains V_*L*_ and C_*L*_) and (B) Heavy Chain Only Antibodies (HCAbs), schematic representation with domains V _H_H, C_H_2 and C_H_3; (C) 2D representation of Immunoglobulin fold of V _H_H domain with demarcated Framework Regions (FRs) and Complementarity Determining Regions (CDRs), loop lengths are approximated for representation purposes. FR1 is composed of β-strands A1, A2 and B, FR2 is composed of β-strands C and C′, FR3 of 4 β-strands C″, D, E and F, FR4 end the V _H_H sequence by last β-strand, G. (D) 3D cartoon representation of V_H_H (PDB ID: 1BZQ chain K (Decanniere et al., 1999) and (E) 90° rotation of the same with the CDRs coloured.

Immunoglobulin Gamma (IgG) (see Fig. 1A) is a major component of the immune system in vertebrates. Members of the family Camelidae have a modified form of IgG, called Heavy Chain only Antibodies (HCAbs). HCAbs, as their name suggests, are completely devoid of (i) light chains and (ii) C_H_1 domain in the heavy chain (see Fig. 1B) (Hamers-Casterman et al., 1993). Interestingly, the N-terminal domain of each chain of HCAb, named V_H_H, is functional when expressed independently. A V_H_H domain is 20 times smaller than complete IgG, ranging from 120 to 150 amino acids in length. Each V_H_H domain (see Figs. 1C–1E) has only 3 Complementary Determining Regions (CDR) to bind to their antigens. These loops connect the more structured regions called the Framework Regions (FR), considered sequentially and structurally well conserved.

Due to the lack of a light chain variable domain (V_L_), V_H_H sequences have evolved to adapt to the hydrophilic environment (Muyldermans et al., 1994), also leading to higher thermal tolerance (Van der Linden et al., 1999; Stefan et al., 2002). Additional topological advantages of V_H_H include convex shaped CDR3 found in V_H_H that bind to enzymes (Muyldermans, 2013), to access epitopes which are otherwise inaccessible to classical antibodies. The small size, ease of expression, unique biochemical and biophysical properties of these domains made them (Nabuurs et al., 2012) appealing tool for applied biotechnology, healthcare therapeutics and diagnostics. For instance, V_H_H domains can cross the blood–brain barrier (Rutgers et al., 2011; Nabuurs et al., 2012) or penetrate tumour core, helping in non-invasive screening of tumours (Massa et al., 2014; Rashidian et al., 2015). AbLynx^®^ (Ablynx, 2016) had developed a V_H_H against acquired Thrombotic Thrombocytopenic Purpura which was successful in clinical trials (Peyvandi et al., 2016), and is patented under the name Caplacizumab in Europe.

In their 2017 study, Zuo and co-workers (Zuo et al., 2017) found more than 2,300 sequences of V_H_H domains, but 90% in patents and only 74% in PDB structures. With the availability of huge numbers of V_H_H sequences in the large majority of the proteins, it is interesting and essential to predict models of V_H_H domains, to understand their binding and specific properties. In 2010, our lab proposed the first structural model using comparative modelling of a V_H_H have been designed against ACKR1 (Smolarek et al., 2010b). Since our study, 21 investigations have used *in silico* structure prediction of V_H_H, to either understand structural features or to understand the interactions between V_H_H and its ligand using the molecular docking summarised in Table S1.

The most common approach to protein 3D structure prediction using template-based modelling is comparative modelling. The principle is to build the structural model of a query protein sequence using the structure of a homologous protein as a template. In case of comparative modelling of variable domains of IgGs, peculiar challenges arise, which are: (i) the presence of interspersed amino acid regions with varying sequence conservation, (ii) hypervariable regions showing high diversity in length and in conformation and (iii) prediction of the inclination angle between the V_H_ and the V_L_ domains which determines the antigen-binding interface. For V_H_H, the third criterion does not apply, but the CDR3 loop is longer and is more conformationally diverse compared to its V_H_ counterparts.

Antibody Modelling Assessment group (AMA) has held two assessment meetings in 2011 and 2014 to rank different methodologies such as the Dassault Systèmes BIOVIA (Fasnacht et al., 2014), RosettaAntibody (Sircar, Kim & Gray, 2009), Schrödinger^®^(Beard et al., 2013; Zhu et al., 2014; Salam et al., 2014), PIGS (Marcatili, Rosi & Tramontano, 2008) and KotaiAntibody (Yamashita et al., 2014). Most of them use hybrid modelling, which is to model CDR loops separately (by comparative modelling or ab-intio) and using comparative modelling for FR. Since only V_H_/V_L_ from IgGs were used, the protocols tested by AMA may not be extrapolated to V_H_H structure prediction. Although some of these methods are capable of modelling V_H_H, most of the V_H_H structures in our survey used classical comparative modelling protocols.

Another important point is that V_H_H topology may seem simple with a conserved immunoglobulin fold composed of “conserved” FRs and a reduced number of CDRs; the complexity arising due to longer CDR1 and CDR3 contribute to non-trivial task of structure prediction. We have shown the crucial choice of the structural template (Smolarek et al., 2010a) that can lead to a totally different orientation of the CDRs. No study has summarised the different approaches since our study, or possible limitations and biases. Our work is intended to make a contribution in this area. The present paper provides an update on (i) different sources of information on V_H_H sequence and structures, (ii) global and local properties of V_H_H sequences and structures, (iii) different methods used in 3D structure prediction, and (iv) drawbacks of the most frequently used prediction protocol. The main goal of this paper is to provide useful findings to researchers interested in analysing V_H_H domains and its structure prediction.

## Materials and Methods

### Datasets

V_H_H (nanobody)sequences were obtained from NCBI Genbank (Benson et al., 2005) (https://www.ncbi.nlm.nih.gov/genbank/) and Uniprot (Boutet et al., 2016) (http://www.uniprot.org/). V_H_H structures were taken from the Protein Databank (PDB, https://www.rcsb.org/pdb/home/home.do) (Berman et al., 2000). This dataset was put together by March, 2018. Sequences were aligned using Clustal Omega v1.2.1 (http://www.clustal.org) (Sievers et al., 2011) and using Jalview v.2.10 (http://www.jalview.org) (Waterhouse et al., 2009), and analysed using WebLogo (Schneider & Stephens, 1990; Crooks et al., 2004). CDRs were analysed using PyIgClassify classification (http://dunbrack2.fccc.edu/pyigclassify/) (Adolf-Bryfogle et al., 2015) (described in greater detail below).

### Comparative modelling of V_H_H

Modeller.9v.16 was used in the study to model V_H_H. Briefly, an alignment file in the prescribed format of the query with the desired template(s) is generated. From this alignment, the algorithm derives spatial restraints or probability density functions for each residue to be modelled. 3D model(s) of the query protein are obtained with the optimisation of the input restraints. The best structural model is selected with best DOPE score (Shen & Sali, 2006; Melo & Sali, 2007).

In the study case, two different scenarios were used: (i) 4 templates were used to propose a multi-template approach and (ii) individual templates were used, leading to five distinct cases. In each case a total of 100 models were generated and were ranked using DOPE score.

### Assessment of structural similarity and disulphide bridge conformations of V_H_H domains

The most popular method to evaluate similarity is the distanc-based similarity measure Root Mean Squared Deviation (RMSD); it is calculated by the averaging of distances between *n*-pairs of equivalent atoms. It can be applied to a whole protein or a subset of specified equivalent atoms from two proteins. It must be noted that RMSD value takes only into account equivalent residue, i.e., it is not appropriate when the segments are of different lengths. RMSD was computed with ProFit (http://www.bioinf.org.uk/programs/profit/) that is based on the McLachlan algorithm (McLachlan & IUCr, 1982) and iPBA (http://www.dsimb.inserm.fr/dsimb_tools/ipba/) (Gelly et al., 2011); this last also provides Global Distance Test Total Score (GDT-TS) values (McLachlan & IUCr, 1982; Zemla, 2003).

A recent classification of disulphide bridges (Schmidt, Ho & Hogg, 2006) includes 20 kinds of disulphide bonds based on the signs of the five dihedral angles between the Cα atoms of any two cysteines involved and the Dihedral strain energy. For example, the sign pattern ‘- - - - - -’ indicates that all the dihedral angles *χ*1, *χ*2, *χ*3, *χ* ‘1, *χ* ‘2 between the two C *α* atoms Cys and Cys’ have negative values.

### Local conformational analysis

Secondary structures were assigned with the most widely used algorithm, namely DSSP (CMBI version 2000) with default parameters (Kabsch et Sander 83). Protein Blocks (PBs) were also used. PBs are a structural alphabet composed of 16 local prototypes (Joseph et al., 2010) five residues in length. PBs give a reasonable approximation of all local protein 3D structures (de Brevern, Etchebest & Hazout, 2000) and are very efficient in protein superimpositions (Joseph, Srinivasan & de Brevern, 2012) and MD (Molecular Dynamics) analyses (de Brevern et al., 2005). They are labelled from *a* to *p*. PBs *m* and *d* can be roughly described as prototypes for *α*-helix and central β-strand, respectively. PBs *a* to *c* primarily represent β-strand N-caps and PBs *e* and *f* represent β-strand C-caps; PBs *g* to *j* are specific to coils; PBs *k* and *l* to *α*-helix N-caps, while PBs *n* to *p* to *α*-helix C-caps. PB (de Brevern, 2005) assignment was carried out using our PBxplore tool (available at GitHub) (Barnoud et al., 2017).

The equivalent number of PBs (*N*_*eq*_) is a statistical measurement similar to an entropy index. It represents the average number of PBs for a residue at a given position which is calculated as follows (de Brevern, Etchebest & Hazout, 2000):

(1) \begin{eqnarray*}{N}_{eq}=\exp \nolimits \left( -\sum _{x=1}^{16}{f}_{x}In{f}_{x} \right) \end{eqnarray*}

Where, *f*_*x*_ is the probability of PB *x*. A *N*_*eq*_ value of 1 indicates that only one type of PB is observed, while a value of 16 is equivalent to a random distribution.

To detect a change in PBs profile, a Δ*PB* value was calculated. It corresponds to the absolute sum of the differences for each PB between the probabilities of a PB *x* being present in the first and the second structures (*x* goes from PB *a* to PB *p*). ΔPB is calculated as follows:

(2) \begin{eqnarray*}\Delta PB=\sum _{x=1}^{16}{|}({f}_{x}^{1}-{f}_{x}^{4}){|}.\end{eqnarray*}

Where, *f*^1^_*x*_ and *f*^2^_*x*_ are the percentages of occurrence of a PB *x* in the analysed structures. A value of 0 indicates perfect PB identity, while a score of 2 indicates a total difference.

The change in PB entropy at given position between two different sets of structures analysed is denoted using Δ*N*_*eq*_ which is calculated as the following:

(3) \begin{eqnarray*}\Delta {N}_{eq}={|}{N}_{eq1}-{N}_{eq2}{|}.\end{eqnarray*}

### PyIgClassify database

CDRs support most of the interactions with the epitope. Different classifications have been proposed to analyse them (Chothia & Lesk, 1987; Martin & Thornton, 1996; Al-Lazikani, Lesk & Chothia, 1997; Shirai, Kidera & Nakamura, 1999). The most recent classification of CDRs was proposed by North and co-workers (North, Lehmann & Dunbrack, 2011). The AHo numbering scheme (Honegger & Plückthun, 2001) was used by the authors to enumerate the residues in variable domains. The clusters are regularly updated and are made available through the PyIgClassify database (http://dunbrack2.fccc.edu/PyIgClassify/) (Adolf-Bryfogle et al., 2015). All the CDRs except CDRH3 are included in the classification. The classified CDR loop conformations are based on the (a) type of CDR, (b) length of the CDR loop, and (c) affinity-propagation clustering method (Frey & Dueck, 2007), using a dihedral angle distance function was used to group CDRs. The clusters are grouped into three categories, (1) Type I, i.e., one cluster CDR-lengths, (2) Type II, i.e., predictable CDR-lengths, and (3) Type III, i.e., unpredictable CDR-lengths. According to the authors, CDR H1 (i.e., CDR1 for V_H_H) falls into the Type II category, where the CDR-length combination has multiple possible structures. CDR H2 (i.e., CDR2 for V_H_H) falls under Type I, the one-cluster CDR-length. Since Protein Blocks are descriptors of local backbone conformations, they have also been used to analyse the conformations of dense clusters, which include V_H_H.

## Results

### Sequence datasets

Uniprot contains a limited number of V_H_H annotated sequences (only 9), while 245 are retrieved from Genbank. In the latter, the majority of V_H_H domains (233) are from Camelids. In the PDB, 140 V_H_H structures were available at the time of dataset generation. Sequences from PDB and Genbank were combined to constitute a master dataset of 373 sequences. After removing the redundant sequences at >95% sequence identity, 325 representative sequences were selected (see Dataset S1). It is the largest sequence dataset ever used to analyse V_H_H domains. Our study focuses on V_H_H domains, since others have compared a more limited number of sequences (90) and always compare with V_H_ as a priority (Mitchell & Colwell, 2018b).

### Multiple sequence alignment

**Figure 2:**
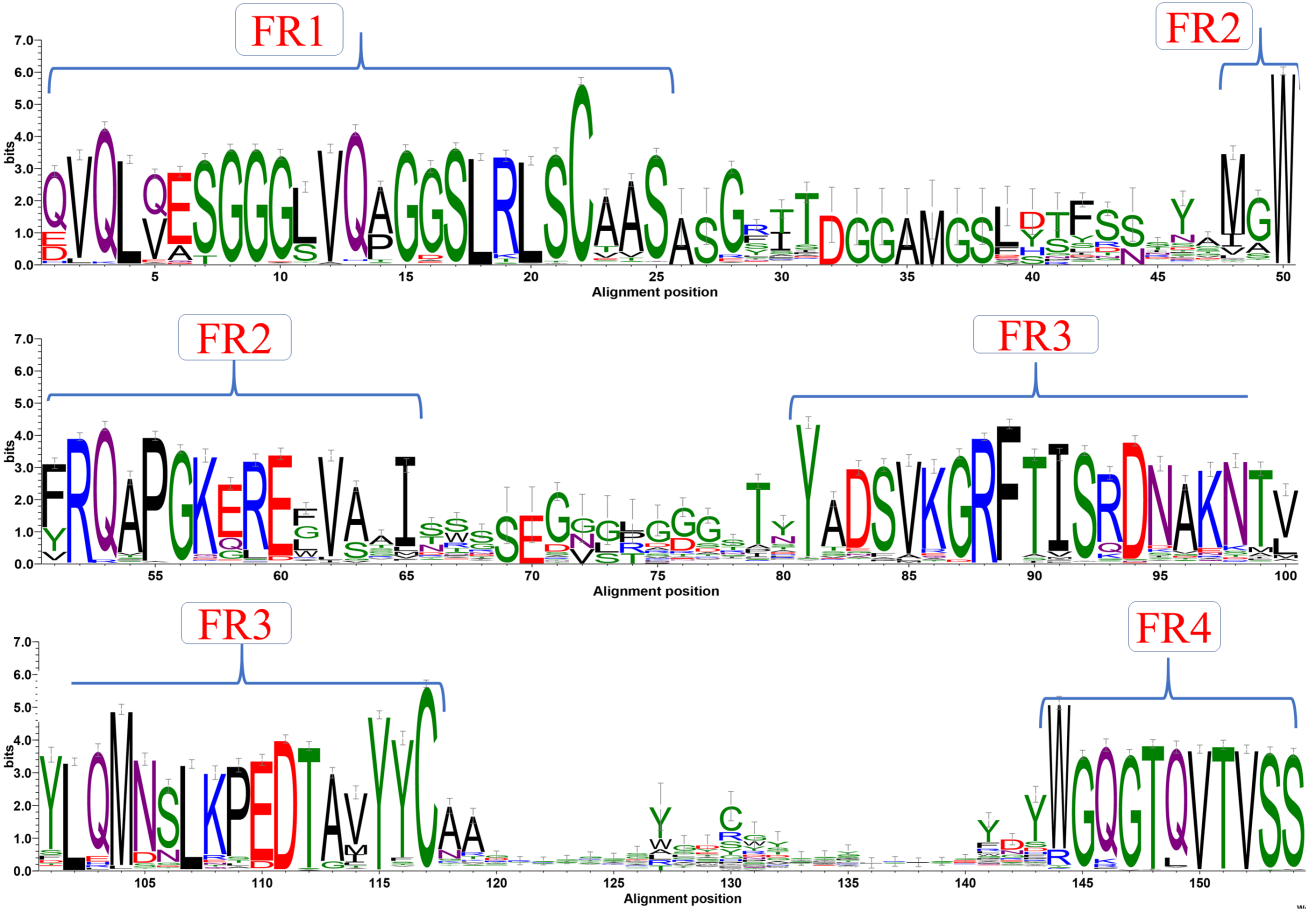
Sequence conservation. Sequence logo representation of multiple sequence alignment of complete dataset of V_H_H sequences. The relative frequency of amino acids at each position is shown here as sequence logo. The residues are colour coded according to their chemical properties. The residue positions are not in accordance with the numbering systems, sequence alignment creates a longer length than canonical V_H_.

A first step in the analysis of a specific protein family is to look at amino acid sequence characteristics using a multiple sequence alignment (MSA). Such an alignment was generated with sequence dataset using Clustal Omega (see Fig. S1). Figure 2 shows the analysis of this MSA represented as a sequence logo, where residue conservation at each position was calculated as information content (bits). As expected, FR positions strikingly appear as conserved sequence blocks evidenced by high bit scores. The interspersed CDRs, on the other hand, have less information content in terms of bits. This is mainly due to inherent sequence variability. Results are in good correspondence with the recent analyses of Mitchell & Colwell (2018a), but show more information in terms of bits for the CDRs than this study. This can be due to the use of an alignment tool that can provide less efficient alignment in variable regions, or simply to a larger number of analysed V_H_H domains, since both free and complexed forms are studied. For the first time, this analysis was also performed for each genus (see Fig. S2 for a summary and Figs. S4–S5 for complete sequence profiles). Interestingly, even with the divergence of species, sequence conservation also shows similar conservation for all the different regions with no significant differences.

V_H_H sequences have a median length value of 123 amino acids (aa) with minimal and maximal length of 109 aa and 137 aa, respectively (see Fig. S6). The amino acid length distribution in different regions of V_H_H (see Fig. S7) shows diversity in CDR lengths, especially in CDR3. The median values for CDR lengths are 8 aa, 8 aa and 16 aa for CDR1, CDR2 and CDR3, respectively. The average length of CDR3 in V_H_H is higher than conventional human or mouse V_H_ sequences (Muyldermans et al., 2009). However, an important point is that FRs are not of absolute invariant length: FR1 is 25 aa in length, FR2 is 18 aa, and FR3 is 37 aa, but with some differences of 2–3 residues. These differences are often forgotten, i.e., in the recent (Mitchell & Colwell, 2018a; Mitchell & Colwell, 2018b) studies, but must be taken into account, or else the analyses could be biased.

The pairwise sequence identity between sequences in the dataset has a median value of 62% and is always above 35% (see Fig. S8A). Thus, it can be considered a relevant case for homology modelling. The variability of amino acids is not constant in these domains. FRs are more conserved, with sequence identity 84, 72, 81 and 90% (median values) for FRs 1, 2, 3 and 4, respectively (see Figs. S8E–S8H). In the case of CDRs, low sequence identities are observed (below 30%); CDR3 is the lowest with 18%, followed by CDR2 with 25% and CDR1 with 28% (see Figs. S8B–S8D). These results also underline why it is so important to keep a high sequence threshold for building of the dataset, since FRs are highly redundant. Hence, a threshold of 30% would have selected only one V_H_H.

### Sequence characteristics

At the time of their discovery, V_H_H sequences were known to have unique amino acid substitutions in FR2 (Vu et al., 1997) compared to V_H_ found in camelids at positions V42F/Y, G49E/K, L50R/C, W52G/L (IMGT^®^ numbering scheme, see Fig. S1). It was thought that there is a single type of V_H_H germline sequence, which has sequence similarity to Clan III of V_H_germline sequences from humans. However, the discovery of V_H_H without this tetrad added exceptions to the former theory. These V_H_H domains are assumed to be derived from a different clan similar to Clan II of V_H_ of human germline sequences (Deschacht et al., 2010), and represent 23 sequences (8 from PDB and 15 from NCBI Genbank). Hence this new analysis underlines that the amino acid substitutions of V_H_H of FR1 and FR2 are entirely non-specific: (a) V(F/Y) also has V at the alignment position 51 in Fig 2.3, in 20% of cases, (b) in G(E/K), E is majority, but K is not found in our dataset and D can be also found (no G at all) at position 58, (c) in L(R), R is majority at position 59, but in a few cases L is found, like for classical V_H_, and finally (d) W(G/L) alignment position 61 is explicit, since G and L represent 50% of the residues while F represents the rest, with V_H_ typically another aromatic W. It is the same thing with L11S, where S is found in 1/3 of the cases and the rest is L –supposedly not typical.

A second kind of unique characteristics are those of the Cystine residues. The conserved Cys23 (FR1)/Cys104 (FR3) disulphide bond is observed in all the variable regions irrespective of the type of chain or species (highlighted in green in Figs. S1). Some of the camelid V_H_H sequences are known to possess an additional disulphide bond between CDR3 or CDR1, CDR3 or CDR2/FR2. In sequences from llamas and camels (see Figs. S3, S4) the presence of an extra cysteine in CDR3 is compensated by another cysteine in CDR1 or CDR2 or CDR2/FR2 boundary. In case of V_H_H from llama, 8 out of 68 structures from PDB show this signature, whereas in camels this number is slightly higher, with 15 out of 34 structures from PDB. In the case of V_H_H sequences from alpacas, this extra cysteine bond is formed between cysteines of CDR3 and FR2/CDR2 boundary with 3 out of 11 structures from PDB (see Figs. S5).

**Figure 3:**
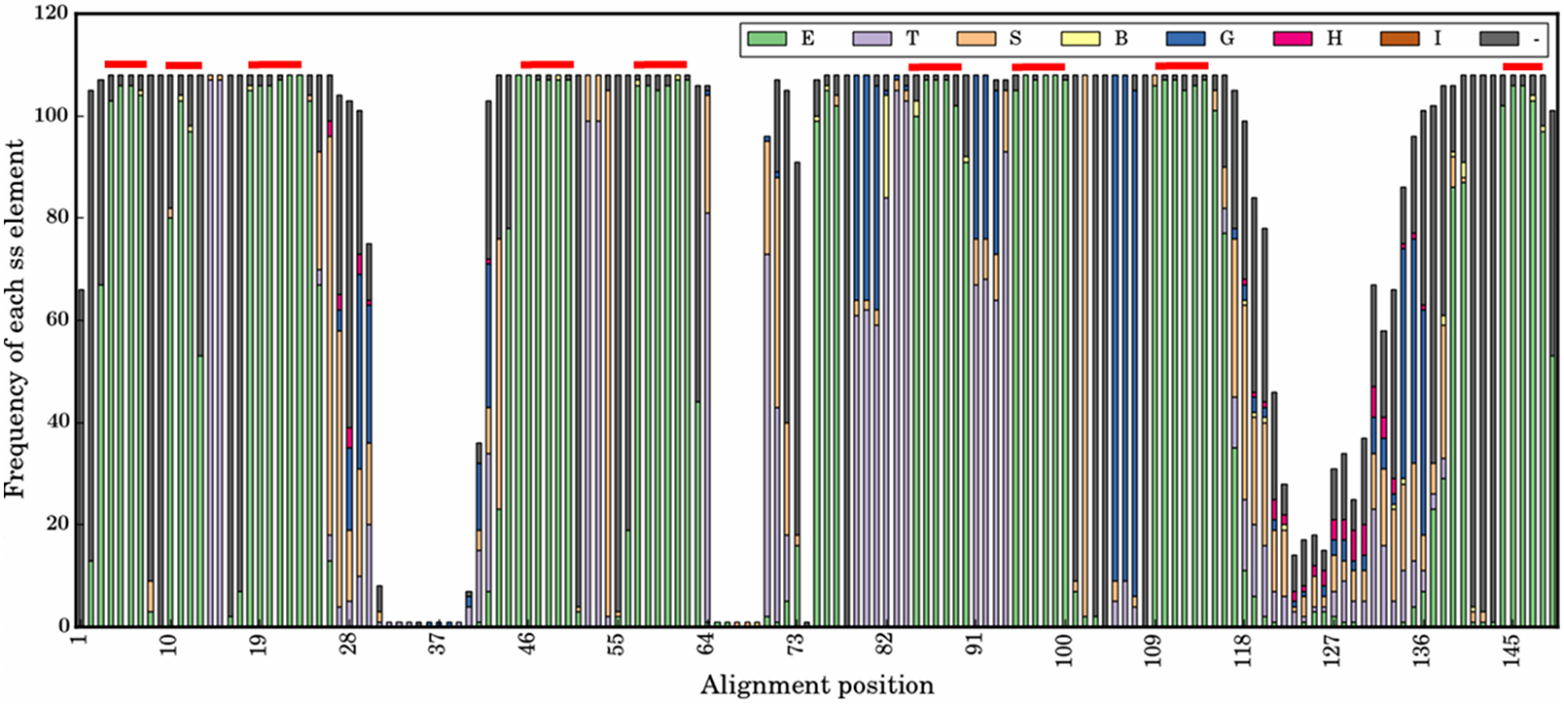
Secondary structural profile of the 105 V _H_H domains. The eight classes of DSSP elements are colour coded in the Figure legend. The symbols are E for extended conformation, (b-strand from b-sheet, T for hydrogen bond turn, S for bend, B for isolated b-bridge, G for 3_10_-helix, H for a-helix, I for p-helix, and ‘-.’ for coils. The different β-strands that form the FRs are indicated above in red: from left to right are the β-strands A, A′, B, C, C′, C″, D, E, and F. The *X*-axis represents the numbering in the alignment.

### Structural analysis of V_H_H domain

As mentioned previously, V_H_H adopts the immunoglobulin fold formed by the anti-parallel arrangement of 9 β-stands connected by loops. Each V_H_H domain has three CDR loops. The fold is held together by the inter strand hydrogen bonds and disulphide bond(s). The conserved disulphide bond is between Cys23 of FR1 and Cys104 of FR3 (IMGT numbering). In few cases the presence of a second disulphide bond is also observed, which is known to increase the overall stability in the respective V_H_H structures (Zabetakis et al., 2014). A non-redundant set of 105 V_H_H domains was assessed for structural domain similarity, using RMSD. The median RMSD value for the whole domain was at 2.63 Å (Figs. S9A–S9H), all the FRs showed a median value <0.8 Å (Figs. S9E to S9H), while they rose to 2.01, 1.56 and 3.51 Å for CDR1, CDR2 and CDR3 respectively (see Figs. S9B to S9D).

### Secondary structure analysis of V_H_H domain

At a more local conformational level, Fig. 3 is a representation of the secondary structures when aligned as per sequence-aligned positions. The nine conserved β-strands (in alignment positions 3–7, 10–13, 18–26, 44–50, 58–62, 85–90, 95–99, 109–119, 144–148) can easily be observed. The CDR regions represented in the alignment at positions 29-41, 64-75 and 116-139, are mainly associated with turns, bends and coil secondary structure elements (SSEs). Interestingly, the connecting loops between some β-strands, encompassed in FRs, are composed of turns, bends and helical SSEs, i.e., positions 13–17 in FR1, 51–59 in FR2, 78–84, 91–94 and 100–108 in the FR3 region. This suggests some of them have different backbone constraints compared to the rest. The middle region of FR4 is an interesting region with almost all coils, which are the irregular SSEs for alignment positions 140–143.

The noteworthy point in the analysis of the different canonical β-strands that are in FRs is that some are close to pure β-strands such as β-strand F (for which it is 99%), but for most of them some positions supposed to be only β-strands are not. They are mainly N-terminus first residue(s). For instance, the first position of β-strand A1 is only 70% for β-strand; it is 80% for β-strand C or A2. The most striking case is the C-terminus of β-strand F for which many residues are either β-strand or β-turns, i.e., clearly different types of local conformations. These results (i) are in concordance with previous analyses using PBs (Noël, Malpertuy & de Brevern, 2016), and (ii) show that FRs are not as conserved in terms of secondary structure, i.e., this finding can so have a direct impact on the proposition of structural models.

### Analysis of disulphide bond geometry in V_H_H domains

**Figure 4:**
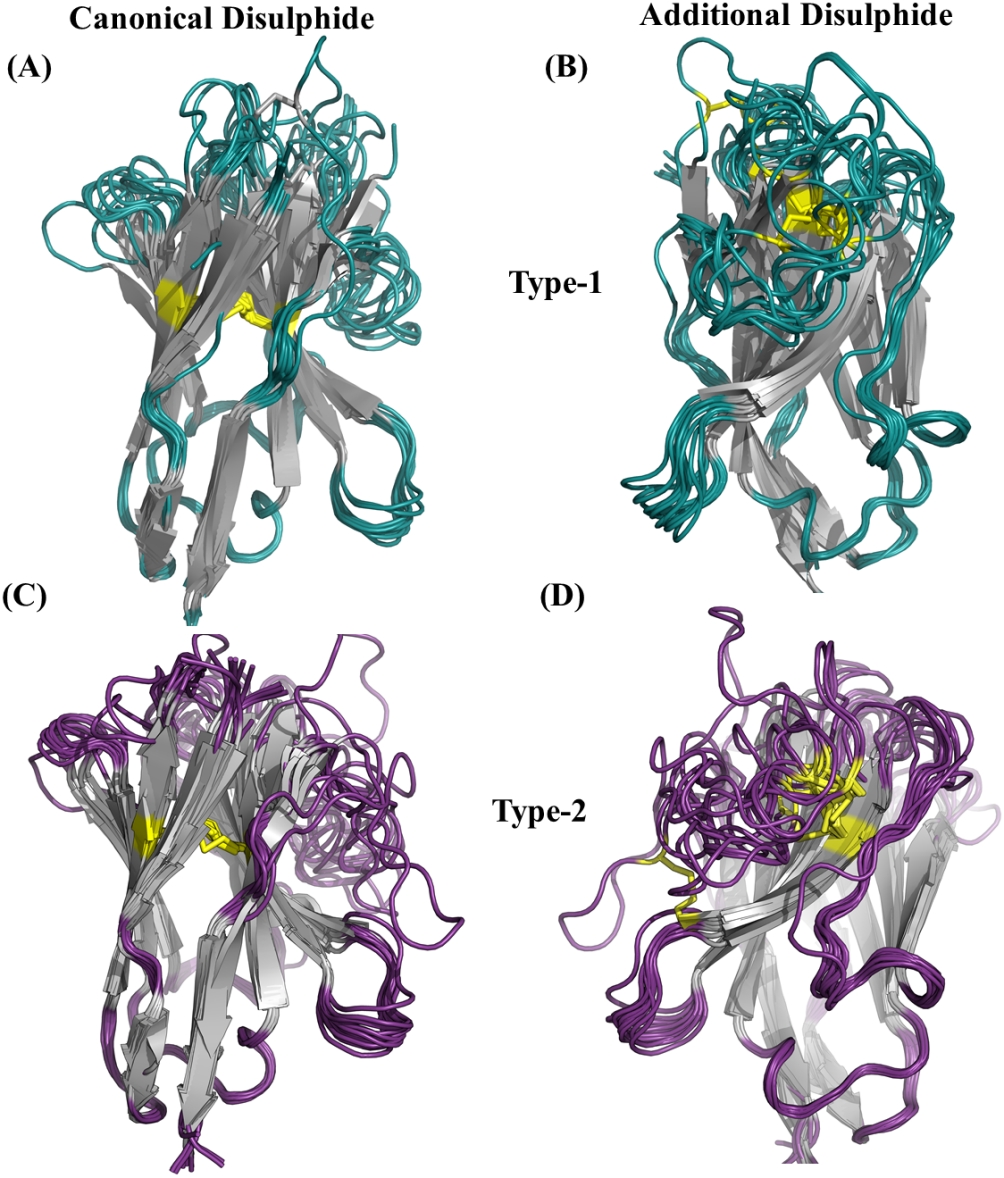
Disulphide bridges of V _H_H domains with additional disulphide bonds. (A) Conserved disulphide bond in type 1 V _H_H domains (B) Non conserved disulphide bonds in type 1 V _H_H domains, (C) Conserved disulphide bond in type 2 V _H_H domains and (D) non-conserved disulphide bonds in type 2 V _H_H domains. The disulphide bridges are indicated in yellow connecting any two cysteines.

An additional disulphide bridge is present in some V_H_H domains along with the conserved-canonical disulphide bond between Cys 23 and Cys 104 (IMGT numbering system). These additional disulphide bridges were observed between Cysteine residues found (i) CDR1 and CDR3, namely type 1, (ii) FR2 and CDR3, namely type 2 and (iii) both in CDR3 (see Figs. S10 to S10C for schematic representation). In our initial dataset, 25 V_H_H domains were observed to have the type 1 (12 V_H_H domains) and type 2 (13 V_H_H domains) additional disulphide bridges. Figure 4 illustrates their position in V_H_H structure, with both types involved in bending CDR3 onto the β-sheet surface.

Using a recent classification of disulphide bridges (Schmidt, Ho & Hogg, 2006) (see Material and Methods section and Figs. S11 for schematic representation) we have analysed the geometry of disulphide bonds in subset of V_H_H structures which contain two disulphide bonds. The sign pattern of the canonical and the additional disulphide bonds of both types have been characterized (see Table S3). The greater the number of negative values a dihedral angle pattern has, the lower is the strain energy, suggestive of a stable disulphide bond. Although, the canonical disulphide bond is conserved in terms of position of cysteines, the sign pattern of the dihedral angles varies much more compared to the additional disulphide bond which is surprising. Interestingly, most of the additional disulphide bonds all have negative sign patterns, suggesting a lower energy bond which might increase stability. Two V_H_H domains (PDB id 1YC8 from type 1 and 4Y7M from type 2 class in Table S3) have the dihedral angle pattern characteristic of allosteric disulphide bonds according to the classification. This analysis shows that both canonical and the additional disulphide bridges do not play a simple structural role, as many are not favourable, and can be or are associated to functional roles of V_H_H domains.

### Analysis of local conformations of CDR1 and CDR2

**Figure 5:**
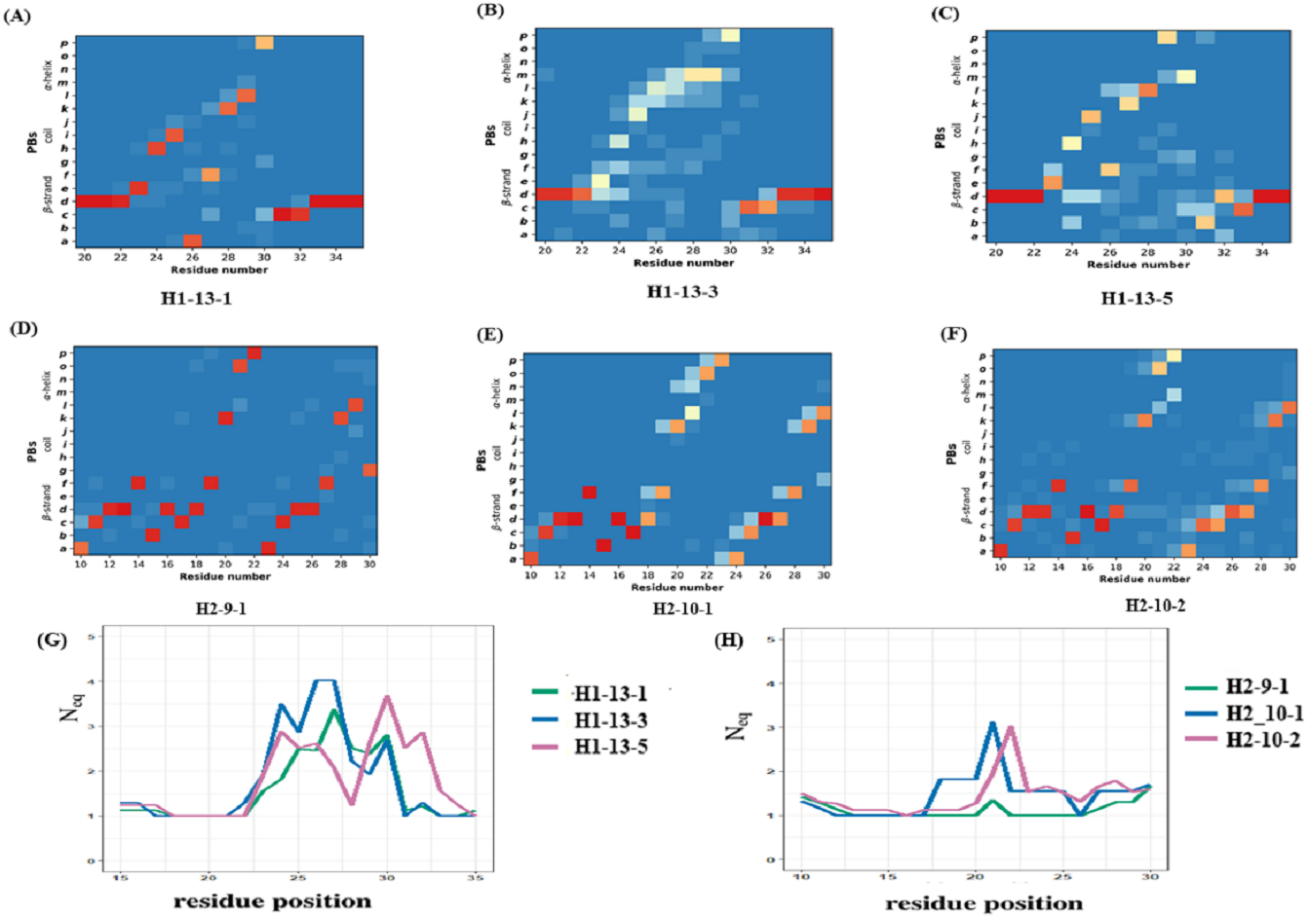
Local conformational analysis of CDR clusters from PyIgClassify. PB maps of CDR H1 region from V _H_H sequences from CDR H1 clusters (A) H1-13-1, (B) H1-13-3, and (C) H1-13-5, CDR H2 region from V _H_H sequences from (D) H2-9-1, (E) H2-10-1, and (F) H2-10-2 cluster, and N_*eq*_ of (G) three CDR H1 clusters and (H) three CDR H2 clusters. The numbering of residues in each plot is according to the IMGT numbering system.

The CDR1 region is defined between the first conserved cysteine (Cys 22) and the conserved tryptophan (Trp 41). The recent PyIgClassify database has 30 clusters of CDRH1 and 19 of these clusters have V_H_H structures. Out of the 19 clusters, 13 are very sparsely populated in V_H_H structures (<10 structures). Clusters H1-13-1, H1-13-3, and H1-13-5 were analysed, as they were associated to a correct number of occurrences (>20 structures). PB analyses of these clusters show variations in PB assignment in the CDR1 region (see PB frequency maps in Figs. 5A to 4C). The residue region from 21 to 33 is the CDR1 according to PyIgClassify. The PB motif includes 3 residues flanking the CDR1, representing the transition from the β-strand region represented by PB *d* to loop and back to β-strand. The first cluster (H1-13-1, see Fig. 5A) has 21 out of 37 (57%) structures sharing a strict common PB signature *ddddehiafklpccdddddd*; the remaining structures of this cluster are close to this PB series as shown with low *N*_*eq*_ (see Fig. 5G). The highest *N*_*eq*_ value for H1-13-1 cluster is not more than 2 for residue position 32, with PB *p* either changing to *c* or *g*. In the other two clusters no strict common PB signature was observed. The third cluster (H1-13-5, see Fig. 5C) has the highest *N*_*eq*_ value. The PBs series of the first cluster *dehiafkl* is replaced by *dehjfklp —* a striking difference*.* The observed structural words in the first cluster PBs *dehia*, *fklpc* and *cdddd* are highly recurrent and reported to have an RMSD below 1 Å, while in the third cluster, the transitions are from the most to the less frequent ones (see the most frequent PB series in de Brevern et al., 2002). As seen in Figs. 5B and 5G, the cluster H1-13-3 has the highest *N*_*eq*_ values and does not show specific PB series like the two other clusters. We can notice small tendencies towards α helical PBs like PB *m*. A similar in-depth analysis was performed for three CDR2 clusters that also show heterogeneity in the three clusters (see Figs. 5D to 5E and Data S1). In summary, the analyses of CDR1 and CDR2 V_H_H in light of PyIgClassify clusters using PBs show a large diversity.

### Inter-cluster comparison

The previous analyses showed the variations within a cluster. Using Δ PB and Δ*N*_*eq*_, it is possible to compare the clusters directly (see ‘Materials and Methods’ section and Fig. S12 to S12D). ΔPB profile allows a detailed description of the conformational diversity between any two sets of protein structural regions. For H1-13-1 and H1-13-5, Δ PB is always higher than 1.5, showing a completely different PB series occurrence. Hence, these two clusters sample two different conformational spaces.

For H2-9-1, H2-10-1 and H2-10-2, ΔPB shows that PB series are closely related in the N-terminal regions between H2-9-1 and H2-10-2, and in C-ter for H2-10-1 and H2-10-2, leading to the idea of composite series with a common PB at position 26 (all ΔPB values of 0.1), information that cannot be provided using RMSD quantification. This analysis shows the relevancy of PBs to compare V_H_H structures and V_H_H structural models in the following sections. It allows a precise comparison of V_H_H structures, which can be considered as highly similar but are not identical, and which quantify this local distance.

### V_H_H structure prediction survey

V_H_H structures are essential to understand binding with partners. Molecular modelling is a simple idea to propose structural models. We performed a complete survey of the V_H_H structure prediction studies published so far (see Table 1). Two main methodologies have been used. (i) generic homology modelling like Modeller and threading approaches like i-TASSER, and (ii) hybrid modelling where FRs and CDRs are modelled separately and then assembled together, like RosettaAntibody (Sircar, Kim & Gray, 2009), ABodyBuilder (Dunbar et al., 2014; Leem et al., 2016), or BioLuminate from Schrödinger^®^ (Beard et al., 2013; Zhu et al., 2014; Salam et al., 2014). Of the above-mentioned approaches, Modeller was used in 50% of the cases in the literature.

All the different structure prediction studies are presented in brief in Table 1 and in detail in Supplemental Information 2). We have presented and classified all the 22 studies into three groups based on the method followed; the reader can, if he wishes, go directly to the following section where we test critically an example of V_H_H structure prediction.

#### (a) V_H_H structure prediction using Modeller

Modeller is the most popular comparative modelling tool known to the community. It is mainly used as a standalone algorithm, but also through specialised servers like Protein Structure Prediction server, PS^2^V^2^ (Chen, Hwang & Yang, 2009) and EsyPred3D (Lambert et al., 2002) or in commercial software like BIOVIA™ (earlier Discovery studio). The standalone Modeller version was used to propose structural models of V_H_H in studies against DARC (Duffy antigen Receptor Chemokine) (Smolarek et al., 2010b), VEGFR2 (Vascular Endothelial Growth Factor Receptor 2) (Shahangian et al., 2015), TNFR1 *α* (Tumour Necrosis Factor Receptor 1 α) (Steeland et al., 2015), PLA_2_ (Phospholipase A2) (Chavanayarn et al., 2012), BMP4 (Bone morphogenic protein 4), MMP8 (Matrix metalloproteinase 8) (Demeestere et al., 2016), PLA_2_ toxins *B. jararacussu* Bothropstoxin-I (BthTX-I) and Bothropstoxin-II (BthTX-II) (Prado et al., 2016). A histone binding V_H_H (Jullien et al., 2016) was proposed through BIOVIA™ suite.

#### (b) Other generic 3D prediction approaches

The fold prediction software Phyre2 was used to model V_H_H against Urease (Hoseinpoor et al., 2014). The popular i-TASSER web server was used for V_H_H designed against PrA (ProteinA) (Fridy et al., 2015) and HCV Non-structural protein NS3/4A (Jittavisutthikul et al., 2015). Raptor-X, another threading-based server, was used to model V_H_H against adenylate cyclase-hemolysin toxin and the repeats in toxin (CyaA-RTX protein) (Malik et al., 2016) subdomains (see Supplemental Information 1).

**Table 1:** Summary of structural modelling studies in chronological order. From left to right, the columns are the names of the authors, year of publication, algorithm of choice for template selection, number of templates used per query, algorithm used for modelling, algorithm used for model validation, algorithm used for model refinement, target/ antigen against which the V_H_H in the study is generated, name of the V_H_H used in the study, organism from which the respective V_H_H is synthesized and PDB ID of the template(s) used in the study.

**Authors**	**Year**	**Template selection**	**Templates/query**	**Modeling methods**	**Antigen**	**VHH (main study)**	**Template(s)**
Smolarek et al.	2010	PSI-BLAST against PDB	one	Modeller	DARC- C terminus	CA52	1OP9 and 1 JTO
Govaert et al.	2011	mutation studies	not applicable	Esypred3D,Robetta for mutants	not applicable	cAbAn33, cAbLys3, and cAbPSA-N7	not mentioned
Sircar et al.	2011	BLAST	one or many	Rosetta Antibody VHH suite	not applicable	not applicable	not applicable
Chavanayarn et al.	2012	BLAST	one	not mentioned	Phospholipase 2 of Naga koultia	P3-1, P3_3	1VHP and 1MVF
Thueng-in et al.	2012	BLAST	one	not mentioned	NS5B(RNA dependent RNA polymerase) of hcv	VHH6, VHH24 (clone names)	1VHP, 1F2X
Phalaphola et al.	2013	BLAST	one	not mentioned	NS3-C ( HCV helicase)	VH6,VHH9,VH59	1OHQ,1XFP,3BN9
Inoue et al.	2013	not mentioned	one	MOE (CCG)	Hen Egg White Lysozyme	cAb-CA05-(C-C-L), cAb- CA05-(#16-#09-L), cAb-CA05-(#16-#19-L)	1RI8
Hoseinpoor et al.	2014	PSI-BLAST ( Phyre2)	one	Phyre2	H.pylori Urease	HMR23	not available
Steeland et al.	2015	not mentioned	multiple	swiss model server	TNF receptor 1	Nb70 and Nb96	4FZE, 4JVP, 3P0G, and 2KH2
Shirin Shahangiana et al.	2015	BLAST against PDB	one	Modelller 9.13	VEGFR	VEvhh1, VEvhh2, VEvhh3	1OP9-A , 1MVF-A and 2X6M-A respectively
Jittavisutthikul et al.	2015	i-TASSER	not mentioned	I-TASSER and ModRefiner	NS3/4A	VHH24, VHH28, VHH41	not mentioned
Unger et al.	2015	BioLuminate suite	multi (different for frs and cdrs of each vhh)	BioLuminate	CDTa/b( clostridium difficile toxin a/b)	1+8, 1-14, 1+18 (3 VHH clones)	numerous
Fridy et al.	2015	based of target- IgG complex PrA1-Fab	one	I-TASSER	not mentioned	LaP-1 to 4	not mentioned
Calpe et al.	2015	BLAST	one	Modeller	Bone Morphogenic factor 4 (BMP)	C4, E7, C8	4BSE, 1SJX
Prado et al.	2016	BLAST against PDB	one	Modeller 9.10	BthTX-I and BthTX-II	KF498607, KF498608, KC329715 and KC329718	4KRP-B, 4DKA-A, 3EZJ-B, 4KRP-B
Jullien et al.	2016	Discovery studio	one	Modeller 9.10 from Accelrys Discovery Studio v. 3.1 (DS 3.1)	Histones 2A and 2B	nabobody against chromatin (Chromatibody)	4IDL
Demeestere et al.	2016	Swiss model web server	multiple	Modeller	Matrix metallo proteinases 8	Nb14	4LAJ, 3EZJ, 3TPK and 4M3J
Se et al.	2016	PS2V2 server	one	Modeller through PS2V2 server	Bap protein Acinetobacter baumanii	VHH1	1MVF
Leem et al.	2016	BLAST	many	Modeller and FREAD (for CDR loop prediction)	not applicable	not applicable	not applicable
Soler et al.	2016	Pre existing knowledge	many	Swiss Model server	lysozyme	NbHuL6, cAbLys, cAbCII10	NbHuL6-3EBA and 3DWT, cAbLys and cAbCII10 1ZMY, 1JTP
Malik et al.	2016	”best fit structures”	one	Raptorx	*CyaA-RTX Segment*	VHH2,VH5,VH18,VHH37	1F2K,4O9H,2KH2,4HEP

#### (c) Antibody specific modelling protocols

Rosetta (Rohl et al., 2004) is one of the two most successful *de novo* approaches. A specialised suite called RosettaAntibody (Sircar, Kim & Gray, 2009) by Gray’s group, was the first to put forward a modelling protocol for V_H_H. The Rosetta Antibody suite was modified to model single chain V_H_H.

Briefly, the templates for FR regions are selected using BLAST against antibody databank containing structures from PDB, and for CDRs the templates are chosen from BLAST bit scores. Once the FRs are modelled, the CDRs are grafted onto the FRs by optimal superposition of the backbone atoms of two overlapping residues at each end of the loop. This study highlights the difficulty in V_H_H modelling, and specifically the fact that an approach for IgG is not optimal for V_H_H. The scripts specific for V_H_H are not available anymore in the newer version of RosettaAntibody, but as suggested by the group, it is possible to model V_H_H by submitting a dummy light chain in the protocol and then deleting it from the models once they are modelled (Sircar et al., 2011).

The most recent class of antibody-specific modelling approaches is ABodyBuilder. Templates are selected using the sequence identity of the FR regions as a criterion against SAbDab (Structural Antibody Database) (Dunbar et al., 2014). Modeller then models FR regions. Next, CDRs are modelled using FREAD, a loop prediction developed by the same group using database search. FREAD basically searches against the CDR database created for each CDR (six in total). The SAbDab is the first database to have text search for querying V_H_H; however, the text search also lists modified V_H_/V_L_ which exist as single domain antibodies (Dunbar et al., 2014; Leem et al., 2016).

### Case study

In case of antibodies, the acceptable range of RMSD is different for CDRs and FRs, often leading to global RMSD <3 Å, but it may not be the only near-native structure/conformation possible (Kufareva & Abagyan, 2012). Improvement of model quality was detailed by using multiple templates (Chakravarty et al., 2004; Larsson et al., 2008). Here, we decided to reproduce the study of Steeland and co-workers (Steeland et al., 2015) to assess the interest and impact of such an approach. They predicted structural models of V_H_H domains (named Nb70 and Nb96) against Tumour Necrosis Factor receptor 1α using multiple templates: (i) Single chain variable fragment (ScFv) from mouse against Interleukin 1 β (PDBID 2KH2 (Wilkinson et al., 2009)), named temp-m), (ii) a llama V_H_H used to stabilize β2 adrenoceptor (PDBID: 3P0G (Rasmussen et al., 2011), referred to as temp-l), (iii) an alpaca V_H_H against Hepatitis C virus (HCV) glycoprotein E2 (PDBID: 4JVP (Tarr et al., 2013), temp-a), and (iv) a V_H_ from human *F*_ab_ which is Antibody-dependent cell-mediated cytotoxicity anti-HIV 1 antibody (PDBID: 4FZE (Tolbert, Wu & Pazgier, 0000), henceforth referred to as temp-h). In the following four sections we will present (a) the analysis of sequence relationship, (b) analysis of the structures, (c) the modelling results and finally (d) how the different structural templates have different impacts on the proposition of structural models.

#### Sequence and structure analysis of templates and query

The query sequence (Nb70) shared a high percent sequence identity (76–70%) with temp-l, temp-m and temp-a (70.6%) and a weak sequence identity (45%) with temp-h (45.3%) (See Table S1A). Hence, three of the structural templates can be considered as good ‘classical’ templates. Amongst them, the first three templates have 67–61% sequence identity, and only 46–37% with temp-h (see Table S1A).

Analysis of FRs and CDRs provided a slightly different view (see Table S1B). Indeed, temp-h still has the worst sequence identity with Nb70 for all FRs and CDRs, except CDR1 (50% sequence identity) and CDR3 (with a sequence identity of only 16%). For the FRs of the others templates, sequence identity reached 96% for FR1, 77% for FR2, 94% for FR3 and even 100% for FR4. For the CDRs, it is lower but remains good, with 50%, 75% and 58%, respectively. Another important point is that CDRs 1 and CDR 2 are of same length in all the sequences (see Fig. S13). The CDR3 region of query is 12 residues long, while temp-a and temp-h are longer, with 20 and 18 aa respectively. These results lead us to conclude that comparative modelling is probably the right choice.

#### Structure analysis of templates

**Figure 6:**
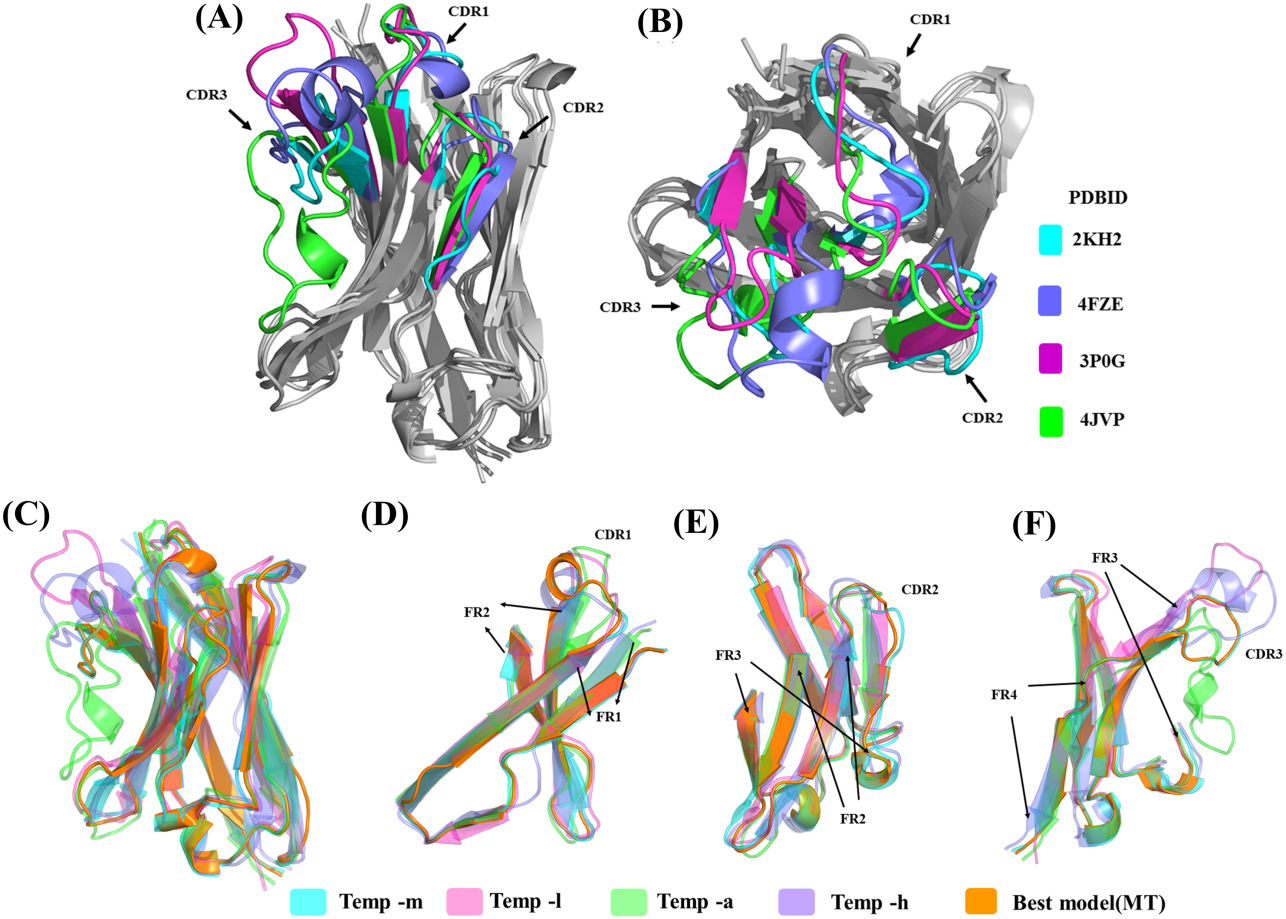
Analyses of structural template and best structural models. First, the four templates Temp -m, Temp -l, Temp -a and Temp -h are superimposed. The colour coded regions in teal, pink, green and violet respectively are the CDRs. (A) Lateral view and (B) top view. Then, best models selected using DOPE score are superimposed. Four templates Temp-m, Temp-l, Temp-a, Temp-h and best structural model colour coded regions in teal, pink, green, violet and orange respectively are shown with different orientations. (A) Global view, (B) zoom on CDR1, FR1 and FR2, (C) on CDR2, FR2, and FR3, and (D) CDR3 with FR3 and FR4.

Sequence similarity from the above analysis suggests good conservation, but structural similarity between the templates provides a different view (see Table S1C). Temp-m is the closest to all three templates, with RMSD values mainly from 1.9 to 2.4 Å, while temp-a is further away with a RMSD value of 3.7 Å with temp-l and of 4.5 Å with temp-h. A large difference between structural templates is observed and does not correspond directly to the information seen in the previous section (see Figs. 6A & 6B). The most conspicuous region in the superposed structures is CDR3 for temp-a and temp-h; in the structure of temp-a, the torso of the CDR3 is bent towards the FR2 region due to the presence of additional disulphide bond. Whereas in the case of temp-h, the CDR3 loop, though long, has a protruding tip (upwards). The RMSD values range from 0.7 to 1.5 Å for FR1, 1.7 to 3.1 Å  for CDR1, 0.4 to 0.9 Å for FR2, 0.3 to 2.2 Å for CDR2, 0.8 to 1.3 Å for FR3, and 0.4 to 1.0 Å for FR4, while CDR3 had segmented values due to difficulty in superimposition. As expected, the general trend of high RMSD in CDRs compared to FRs is observed in all the pairwise comparisons (see Table S2). It is interesting to note the deviations in FR regions between temp-l, temp-a and temp-h, that might be unexpected.

#### (c) Analysis of the structural models

The structural similarity between the best-selected model from multiple template modelling (the reference) and the models generated from single template is shown in Figs. 6C to 6F. The best model constructed using temp-m adopts the conformations similar to that of the best model using multi-templates, especially in the CDR3 region. This is surprising, since it shares a higher sequence identity with temp-l in this region, but CDR3 lengths are the same in both the query and temp-m.

Using residue-wise RMSD comparison as quantification (see Fig. S11), the model generated from temp-m is close to the best model generated from multiple templates, even in the CDR3 region (only the CDR2 region has a significantly higher value). The other best models display much higher residue-wise RMSD in all the CDR regions, even ranging 15 Å in CDR3 for the model generated with temp-a.

Interestingly, one of the structural templates provided a major contribution to the final models, although (i) it does not have a stronger sequence identity than the rest, and even, (ii) the only region for which it had the best sequence identity, CDR2, is the only region that is far from the selected model. Thus, the multi-template approach for V_H_H appears somewhat complex.

#### (d) Analysis of local conformational sampling by Modeller

**Figure 7:**
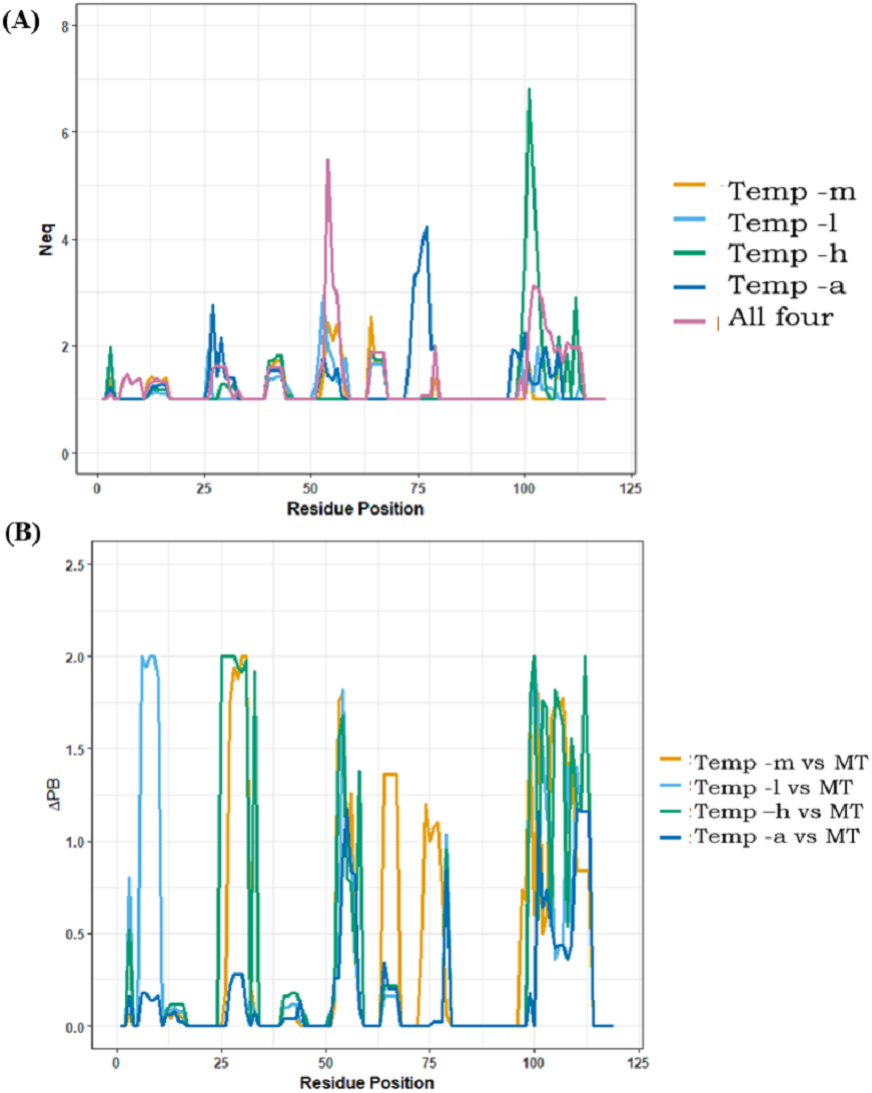
Positional PB entropy N_*eq*_ and ΔPB. (A) All five scenarios of modelling are represented in separate colours. *X*-axis represents residue positions and *Y*-axis represents N_*eq*_. (B) ΔPB between multi-template scenario and each mono template scenario.

Analyses of best models only provided information for best DOPE score selected models; it does not provide information about potential sampling proposed by the comparative modelling approach. Using PBxplore, Protein Blocks were assigned to each of the models and *N*_*eq*_ entropy computed at each position. A summary of *N*_*eq*_ values >1 is provided in Table 2; in all the cases, modelling with multiple templates is the one with the highest number of residue positions with *N*_*eq*_>1 (49 residue positions), suggesting the largest conformational diversity. The least number of residue positions with *N*_*eq*_>1 is for models generated using temp-m (23 residue positions). Notably, models generated from temp-h had two residue positions with high *N*_*eq*_ values in the CDR3 region. Figure 7A is a position-wise distribution of *N*_*eq*_ values in each case. The interesting results are that (i) the *N*_*eq*_ of template temp-m is most often low compared to the multi-template case, and, (ii) the models from multiple templates case show maximum *N*_*eq*_ in the CDR2 region (see also Supplemental Information 2).

**Table 2:** Distribution of *N*_*eq*_ in each modelling scenario.

*N*_***eq***_	**Temp-m**	**Temp-l**	**Temp-a**	**Temp-h**	**All templates**
1–2	19	34	35	23	39
2–3	4	1	3	4	6
3–4			3	2	3
4–5			1		
5–6				1	1
6–7				1	

Analysis of PB distribution (see Fig. S15) shows preferred PB ‘*d* ‘for most residues of FRs in β-sheets in all the cases. The three preceding and succeeding residues of CDRs were considered anchor residues in each loop. Surprisingly, no PB variability was observed for CDR1 anchors. In the case of CDR2, only the position 51 from models generated from temp-l showed a slight variation of *N*_*eq*_ to 1.22. For CDR3, the preceding anchors showed no PB variation; however, the succeeding anchor positions in the modelling scenario of temp-h and temp-a showed variations.

CDRs (aa regions 26–34, 52–58, 97–108), as expected, have more diverse conformations than FRs. Amongst the five scenarios, the most PB diversity in CDR3 is seen in 11 out of 12 residue positions from models generated from temp-a, followed by models generated from temp-l (9/12 residues positions), multiple templates (8/12 residue positions), temp-h (7/12 residue positions) and temp-m (1/12 residue position). This observation suggests that adding more information in the case of multiple templates, and poor template target alignments can cause unexpected conformational diversity.

The local conformational diversity in terms of PBs between mono template(s) and multi-template structural models can be understood by analysing the differences in PBs, quantified by ΔPB. Figure 7B shows ΔPB calculated between multi-template models and each individual template model. Among all four cases of comparison, the case of modelling with temp-m and multi-template shows the least change in PBs at each residue. The differences in local conformations are expected in the CDRs, while the changes in FRs are the ones least expected. These comparisons may also suggest that in case of multi-template modelling, temp-m mostly influenced model conformations due to better alignment quality in CDR regions, and a shared second-best sequence identity with the query. Please note that Nb96 produced roughly the same results, while CDR3 is longer and so more complex to analyse.

## Discussion

Analysis and prediction of simple V_H_H fold seems a trivial task at first sight. Analysis of the sequence content of FRs and CDRs correlates partially with the recent study of Mitchell and Colwell (Mitchell & Colwell, 2018a). They have focused their study on a dataset of V_H_H domain in complex with respective antigens and compared to IgGs. At present, it appears obvious that the expected specific amino acid signature of V_H_H is not a universal feature in these domains and only depends on the germline; few unmodified V_H_H domains lack them. Analysis of V_H_H structures also showed that the fold is far from conserved in terms of local protein conformation. Assignment of secondary structures showed some important deviations, irrespective of the bound or unbound state of the V_H_H. Similarly, analysis of CDRs in light of PBs showed (i) that actual CDR classification is difficult to apply to V_H_H, (ii) use of a global measure in CDR classification tends to associate different types of local protein conformations, i.e., PB series and (iii) in fact, some common PB series can be found in different CDR clusters. Interestingly, the additional disulphide bridges of whatever type are often not strongly favourable, which is slightly counterintuitive at first sight.

A survey of structure prediction studies of V_H_H showed that most studies resorted to generic template-based modelling approaches. Analysis of the impact of template conformations on the model(s) generated, with the example of V_H_H modelled by Steeland and co-workers (Steeland et al., 2015), underlined the difficulty of choosing the template(s). Indeed, three of the four templates have roughly common sequence identity measured at around 74%, each being slightly more identical depending on the CDRs and FRs, i.e., none can be selected as the best of the best. Nonetheless, each of them had a unique 3D conformation and proposed (a) a different best model, but also (b) sampled different conformations. It is, in fact, surprising that the more dissimilar template (45% sequence identity) does not produce V_H_H models distinct from models predicted using other templates. These results clearly indicate the need for more detailed study and approaches to propose optimized methodology for V_H_H structure prediction (see also Supplemental Information 3).

Additionally, we have performed a similar analysis with solved V_H_H structure to confirm our hypothesis. A V_H_H domain (PDBID: 1QD0 (Spinelli et al., 2000) was selected as the query and four other V_H_H sharing sequence identities between 73.9% and 67.5%, representing a real world case. The four templates’ RMSD to the query ranged from 2.01 Å to 4.41 Å. The best structural model obtained using the structural template with highest sequence identity had a 2.1 Å RMSD value. The addition of other templates provided a clear increase to 2.2 Å with two templates, a further increase to 2.6 Å for 4 templates (see Supplementary Analysis 1 and Fig. S16 for more details). The findings corroborate results previously shown in the case study of Nb70 V_H_H domain modelling. These examples taken from the literature are case studies and show how the authors proceeded. They could be improved by a better control of structural templates, or additional constraints such as secondary structures, distances or even Protein Blocks. We underlined that one must pay attention to details of templates such as structural similarity between templates when using multiple templates, and the length of CDRs in addition to sequence identity

## Conclusion

Our paper in addition to reaffirming the sequences-structure characteristics of V_H_H domains reported in the recent literature, also makes unique observations regarding (i) the variation in the amino acid signature in the FR2 region, (ii) conservation of β-strands and presence of other kinds of secondary structures, (iii) sheds light on conformations of di sulphide bridges and (iv) inter and intra cluster variations from PyIgclassify CDR clusters in terms of local conformations using Protein Blocks. All the above analyses might help the community to appreciate the topological enigma of these domains. As Protein Blocks were able to identify many fine variations in the well accepted CDR classification and FRs in our previous paper, we used it to analyse local conformations of modelled structures of V_H_H domain from a study. The variations in local conformations in models are influenced template quality and conformations. In most cases of variable domain comparative modelling, the templates for modelling FRs are selected based only on sequence identity. In the specific case study that we chose, more complexity arose due to the usage of multiple templates which were chosen based on BLAST searches. We intend to draw the attention of the research community by a precise analysis of the models from this exercise to the influences of template in terms of CDR3 length and sequence identity and Protein Blocks sequences. The latter is an innovative approach developed in our lab to shed light on local conformational changes. This exercise suggests that the selected templates might not be the best possible templates when chosen using FR sequence identity. Finally, the use of multiple templates when the templates are overlapping more restraints that may not be desirable.

##  Supplemental Information

10.7717/peerj.8408/supp-1 Dataset S1 V _H_H sequences The sequences of all V _H_H used in the study are provided.

10.7717/peerj.8408/supp-2 Figure S1 Multiple Sequence Alignment profile of V _H_H protein sequences (complete non redundant dataset) Multiple Sequence Alignment is viewed using Jalview. The amino acid positions coloured in violet are positions known to undergo the unique V _H_H substitutions V42F/Y, G49E/K, L50R/C, W52G/L. The two conserved cysteines are coloured in green. The extra cysteines occurring are coloured in red.

10.7717/peerj.8408/supp-3 Figure S2 Residue conservation profiles in Multiple Sequence Alignments of V _H_H from three different genera MSAs of (A) Lama, (B) Camels and (C) Alpaca. All the alignments are viewed using Jalview (Waterhouse et al., 2009). Boxed regions of amino acids in each figure are the Framework regions.

10.7717/peerj.8408/supp-4 Figure S3 Multiple Sequence Alignment profile of V _H_H protein sequences from llama Multiple sequence alignment viewed using Jalview. All cysteines are coloured in red.

10.7717/peerj.8408/supp-5 Figure S4 Multiple Sequence Alignment profile of V _H_H protein sequences from camels See Fig. S3 for legends.

10.7717/peerj.8408/supp-6 Figure S5 Multiple Sequence Alignment profile of V _H_H protein sequences from alpacas See Fig. S3 for legends.

10.7717/peerj.8408/supp-7 Figure S7 Amino acid Sequence length distribution in V _H_H dataset The box plot representation of amino acid sequence length distribution in sequences from total (left) gb (GenBank, centre), pdb (right).

10.7717/peerj.8408/supp-8 Figure S7 Amino acid sequence length distribution in different Framework Regions and Complementarity Determining Regions from the total dataset of V _H_H sequences From left to right are amino acid length distributions in FR1, FR2, FR3, FR4, CDR1, CDR2 and CDR3.

10.7717/peerj.8408/supp-9 Figure S9 Sequence variability in different regions of V _H_H Sequence identities are provided for (A) Complete sequences (median value = 63.5%), (B) CDR1 (median = 28.6%), (C) CDR2 (median = 25.0%), (D) CDR3 (median = 18.0%), (E) FR1 (median = 84.0%), (F) FR2 (median = 72.0%), (G) FR3 (median = 81.0%), (H) FR4 (median = 90.0%). The median values are indicated by the dotted lines.

10.7717/peerj.8408/supp-10 Figure S10 Structural similarity in different regions of V _H_H Average RMSD values are provided for (A) Complete sequences (median value =* 2.63Å), (B) CDR1 (median* =* 2.01 Å), (C) CDR2 (median* =* 1.56 Å), (D) CDR3 (median* =* 3.51 Å), (E) FR1 (median* =* 0.57 Å), (F) FR2 (median* =* 0.58 Å), (G) FR3 (median* =* 0.73 Å), and (H) FR4 (median* =* 0.36 Å).The median values are indicated by the dotted lines.*

10.7717/peerj.8408/supp-11 Figure S11 Three types of V _H_H based on presence of disulphide bridges (A) Additional disulphide bridge is found between cysteines of CDR1 and CDR3, (B) Additional disulphide bridge is found between cysteines of FR2 and CDR3, and (C) Intra CDR3. The FR and CDR definition are according to the IMGT Numbering scheme. The conserved disulphide bridge represented in yellow is formed by C23 and C104; the residue positions of non-conserved cysteines vary.

10.7717/peerj.8408/supp-12 Figure S11 Illustration of disulphide bond geometry Five dihedral angles between the two C *α* atoms of cysteines involved in the bond are represented as *χ*1, *χ*2, *χ*3, *χ*‘1, *χ*‘2.

10.7717/peerj.8408/supp-13 Figure S12 Differences in PB frequencies and entropy between clusters ΔN _*eq*_ is shown in (A) between CDR1 clusters and (B) between CDR2 clusters and ΔPB is shown in (C) between CDR1 clusters (D) between CDR2 clusters. Legends are provided at the left of each plot. Δ*N*_*eq*_** emphasizes a difference between H1-13-1 and H1-13-5 around a value of 1, while between H1-13-1 and H1-13-3 it is above 3. For H2-9-1, H2-10-1 and H2-10-2, profiles are very different, often with Δ*N*_*eq*_** lower than 1 with a common peak around position 23.

10.7717/peerj.8408/supp-14 Figure S13 Sequence alignment of the query V _H_H protein with its templates CDRs 1, 2 and 3 are demarcated according to the IMGT numbering system and coloured in pink, green and red respectively. Below the sequence alignment are the metrics conservation, quality and consensus of the alignment.

10.7717/peerj.8408/supp-15 Figure S14 Residue wise RMSD RMSD between the best structural model of the multiple template scenario and best structural models from individual template scenarios. *X*-axis represents residue position and Y-axis represents RMSD.

10.7717/peerj.8408/supp-16 Figure S15 PB distribution of all five modelling scenarios PB map of models constructed (A) using temp-m (PDB ID 2KH2), (B) temp-l (PDB ID 3P0G), (C) temp-a (PDB ID 4JVP) (D) temp-h 4FZE and (E) using all the four templates. The intensity ranges from 0 to 1, colour coded from blue to red. The *X*-axis on each map represents the residue position; on the Y-axis PBs are shown along with a rough secondary structure association for interpretation.

10.7717/peerj.8408/supp-17 Figure S16 Comparative modelling of V _H_H with a solved crystal structure (A) Multiple sequence alignment of query V _H_H with the selected templates. (B) Structural superimposition of query (yellow), Template-1 (pink), Template-2 (turquoise), Template-3 (green), Template-4 (blue). (C) Structural superimposition of best models in each case with the query (yellow), best model using Template-1 (violet), best model using Templates 1 and 4 (green), using all templates (blue). (D) PB frequency map of structural models generated with Template-1. (E) PB frequency map of structural models generated with Templates 1 and 4, (F) PB frequency map of modelling scenario with four templates, (G) PB entropy at each position for each modelling scenario.

10.7717/peerj.8408/supp-18 Table S1 Sequence and structure comparison between structural templates and query The table provides (A) the sequence identitypercentage between structural templates and query, (B) for each FRs and CDRs (in brackets) it provides the similarity values (the best values are in bold and lowest in italics) and (C) the RMSD between structural templates.

10.7717/peerj.8408/supp-19 Table S2 Root Mean Square Deviation between any two templates in different Framework Regions and Complementarity Determining Regions

10.7717/peerj.8408/supp-20 Table S3 Sign pattern of five dihedral angle (*χ*1, *χ*2, *χ*3, *χ*‘1, *χ*‘2) in canonical and non-canonical disulphide in V _H_H with additional disulphide bridge (A) type 1 and (B) type 2.

10.7717/peerj.8408/supp-21 Data S1 Analysis of local conformations of ** CDR2 of V _H_H**

10.7717/peerj.8408/supp-22 Supplemental Information 1 Detailed description of VHH structure prediction studies

10.7717/peerj.8408/supp-23 Supplemental Information 2 PB variability in CDRs

10.7717/peerj.8408/supp-24 Supplemental Information 3 Conformational changes

## Additional Information and Declarations

### Data Availability

The following information was supplied regarding data availability: The sequences of all VHH used in the study are available as Dataset S1.
