# Supplementary figures and images for: Discrete analysis of camelid variable domains: sequences, structures, and in-silico structure prediction
Source: PeerJ. 2020 Mar 6;8:e8408. doi: 10.7717/peerj.8408 (PMC7061911; doi:10.7717/peerj.8408)

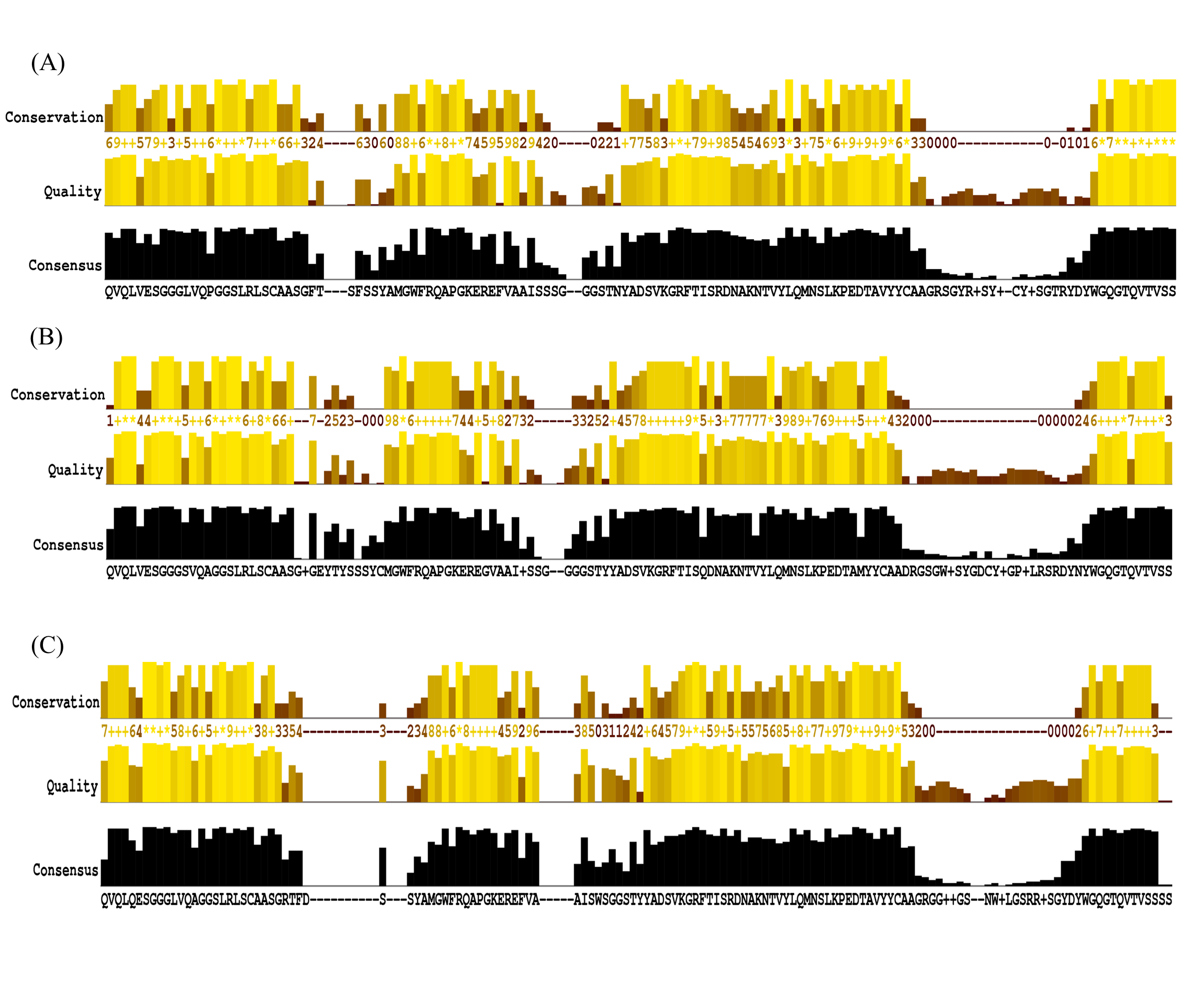

Supplement: Figure S2 — MSAs of (A) Lama, (B) Camels and (C) Alpaca. All the alignments are viewed using Jalview (Waterhouse et al., 2009). Boxed regions of amino acids in each figure are the Framework regions. [file peerj-08-8408-s003.tif]

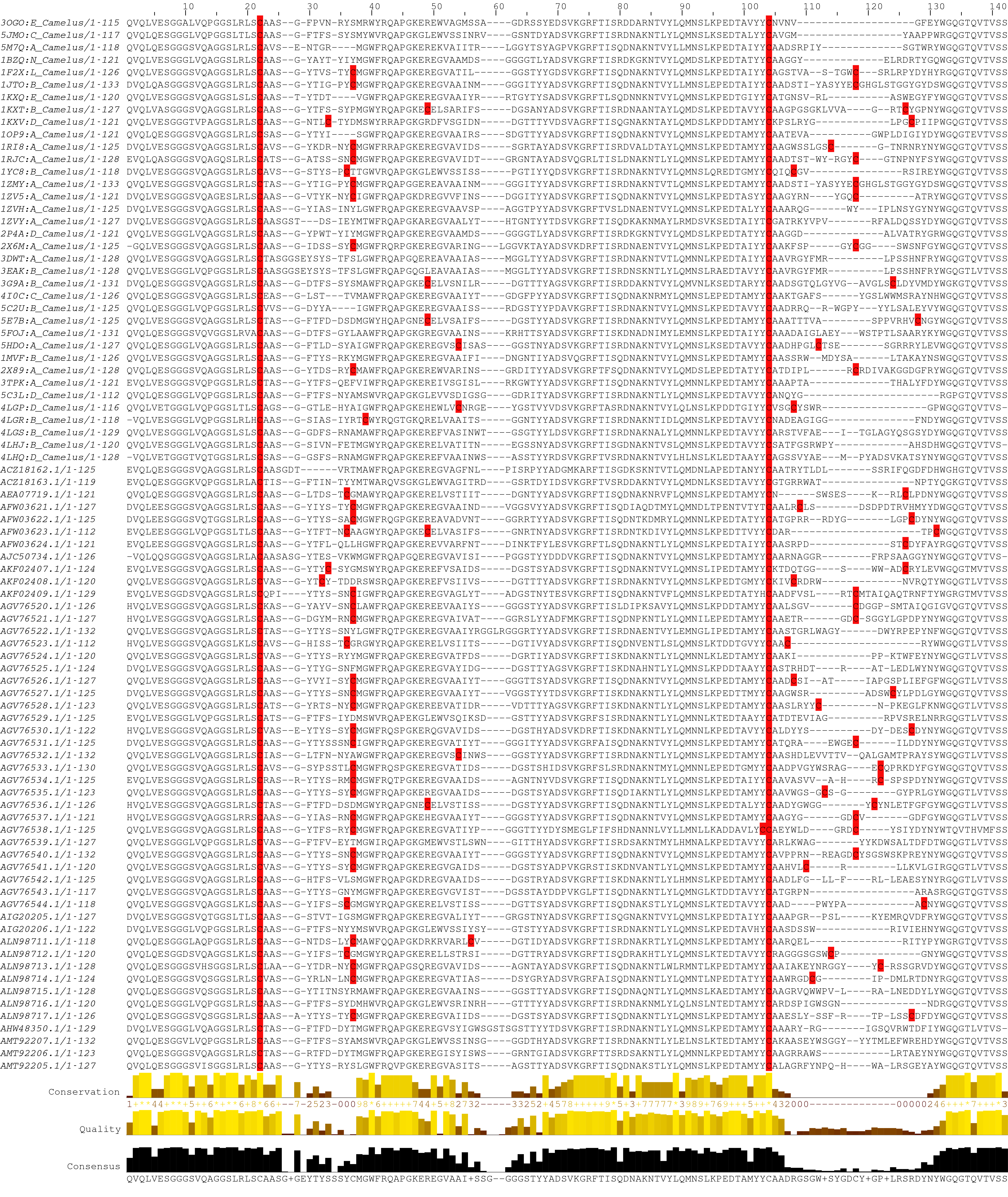

Supplement: Figure S4 — See Fig. S3 for legends. [file peerj-08-8408-s005.tiff]

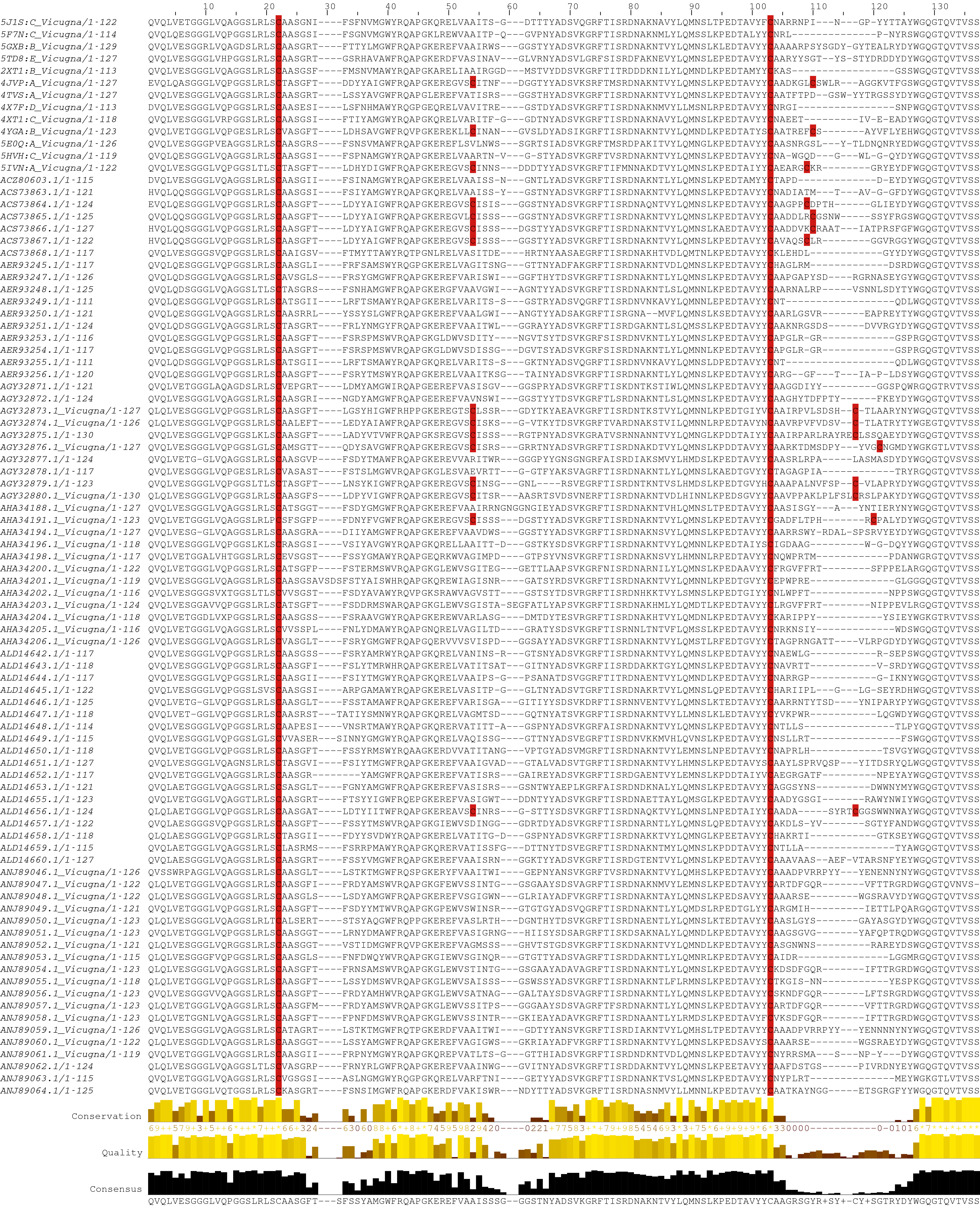

Supplement: Figure S5 — See Fig. S3 for legends. [file peerj-08-8408-s006.tiff]

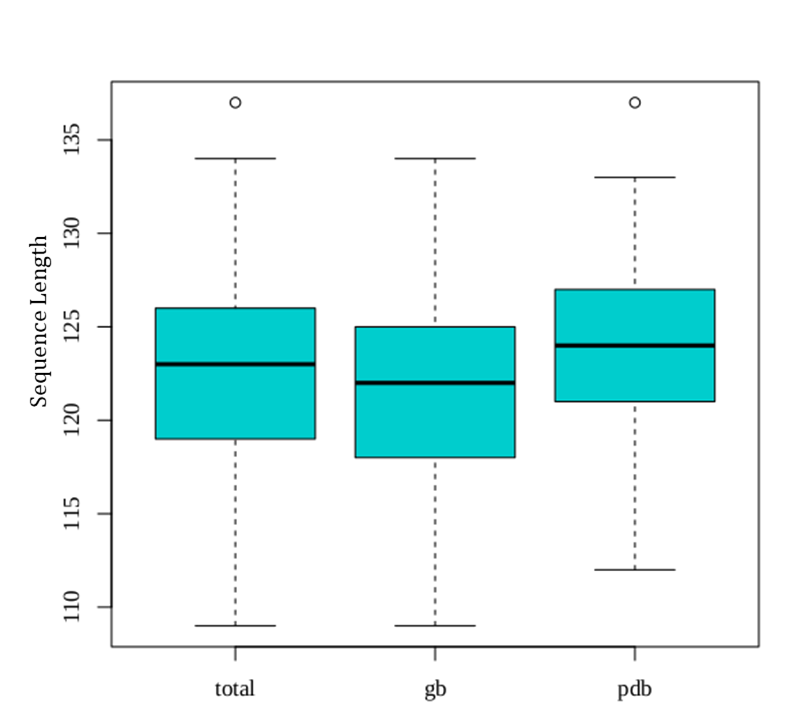

Supplement: Figure S7 — The box plot representation of amino acid sequence length distribution in sequences from total (left) gb (GenBank, centre), pdb (right). [file peerj-08-8408-s007.tif]

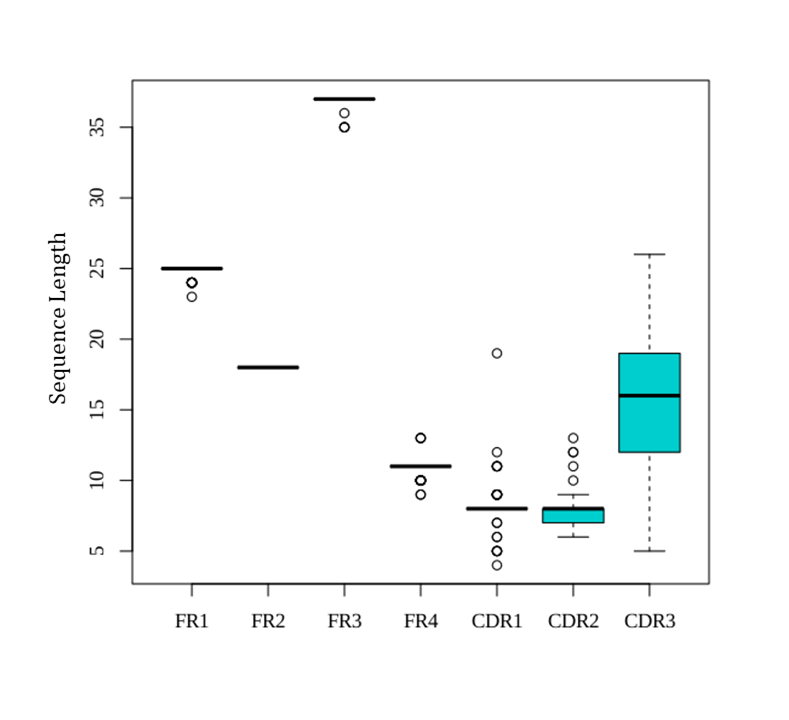

Supplement: Figure S7 — From left to right are amino acid length distributions in FR1, FR2, FR3, FR4, CDR1, CDR2 and CDR3. [file peerj-08-8408-s008.tif]

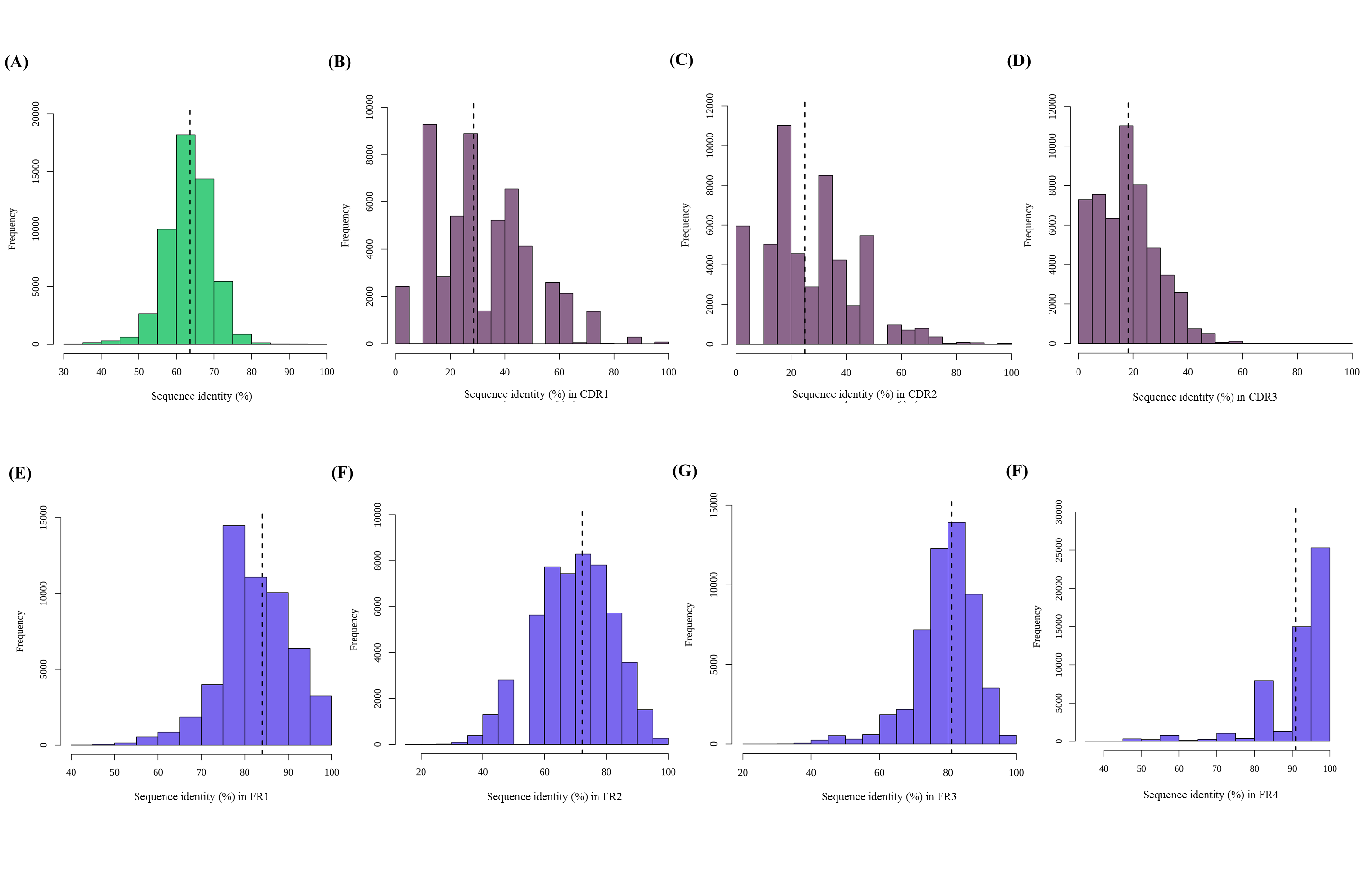

Supplement: Figure S9 — Sequence identities are provided for (A) Complete sequences (median value = 63.5%), (B) CDR1 (median = 28.6%), (C) CDR2 (median = 25.0%), (D) CDR3 (median = 18.0%), (E) FR1 (median = 84.0%), (F) FR2 (median = 72.0%), (G) FR3 (median = 81.0%), (H) FR4 (median = 90.0%). The median values are indicated by the dotted lines. [file peerj-08-8408-s009.tif]

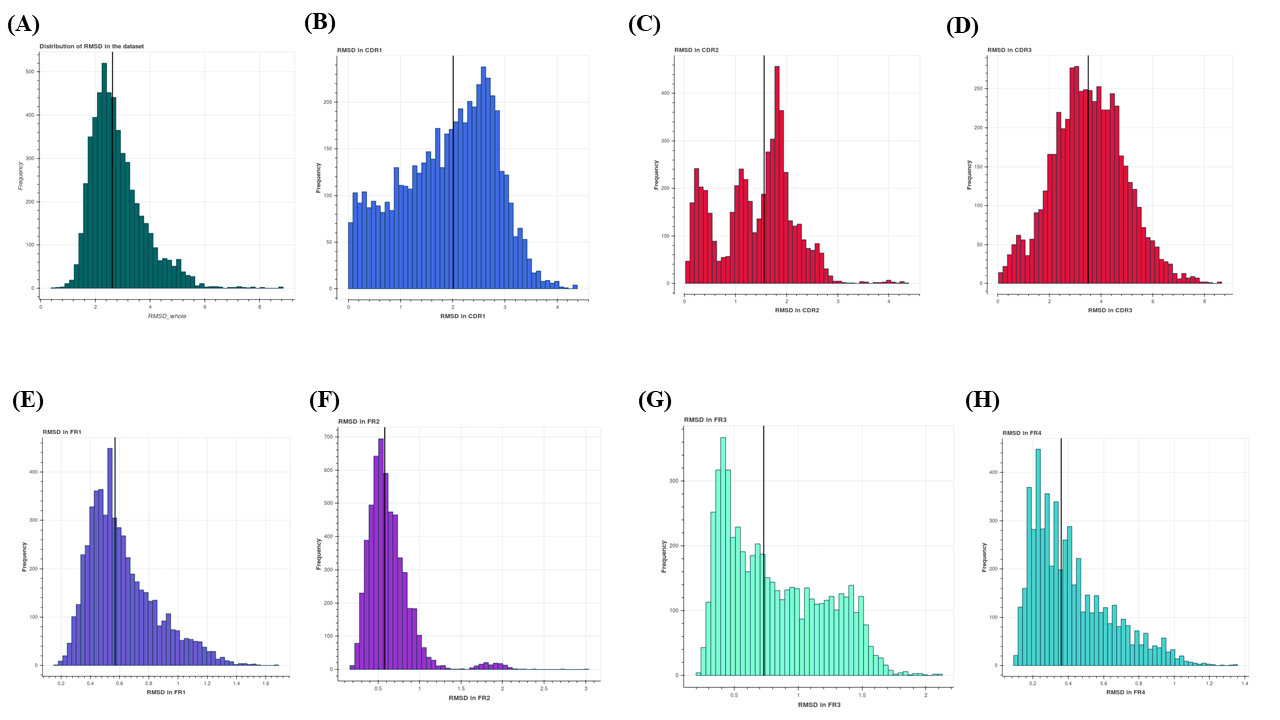

Supplement: Figure S10 — Average RMSD values are provided for (A) Complete sequences (median value = 2.63Å), (B) CDR1 (median = 2.01 Å), (C) CDR2 (median = 1.56 Å), (D) CDR3 (median = 3.51 Å), (E) FR1 (median = 0.57 Å), (F) FR2 (median = 0.58 Å), (G) FR3 (median = 0.73 Å), and (H) FR4 (median = 0.36 Å).The median values are indicated by the dotted lines. [file peerj-08-8408-s010.tif]

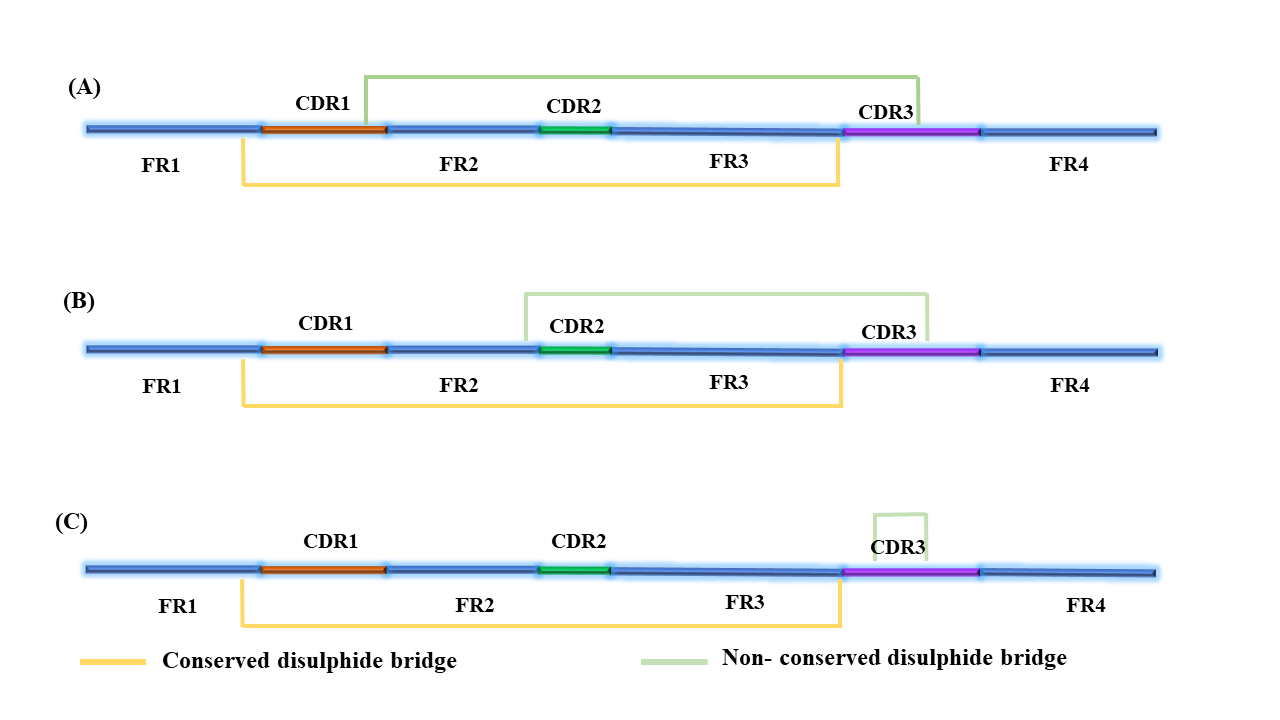

Supplement: Figure S11 — (A) Additional disulphide bridge is found between cysteines of CDR1 and CDR3, (B) Additional disulphide bridge is found between cysteines of FR2 and CDR3, and (C) Intra CDR3. The FR and CDR definition are according to the IMGT Numbering scheme. The conserved disulphide bridge represented in yellow is formed by C23 and C104; the residue positions of non-conserved cysteines vary. [file peerj-08-8408-s011.tif]

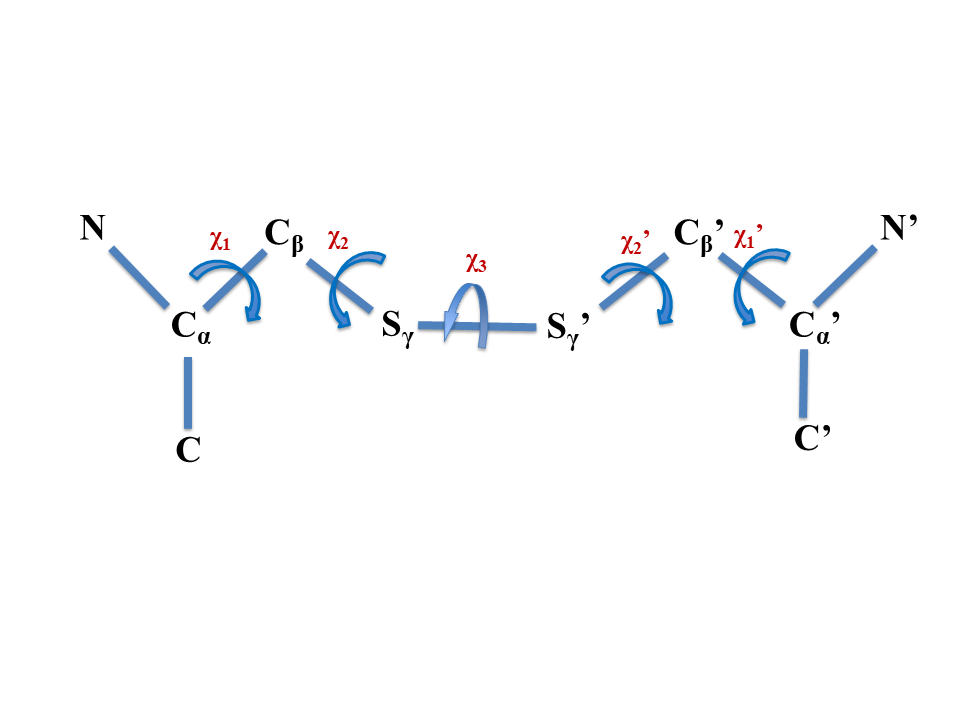

Supplement: Figure S11 — Five dihedral angles between the two C α atoms of cysteines involved in the bond are represented as χ1, χ2, χ3, χ‘1, χ‘2. [file peerj-08-8408-s012.tif]

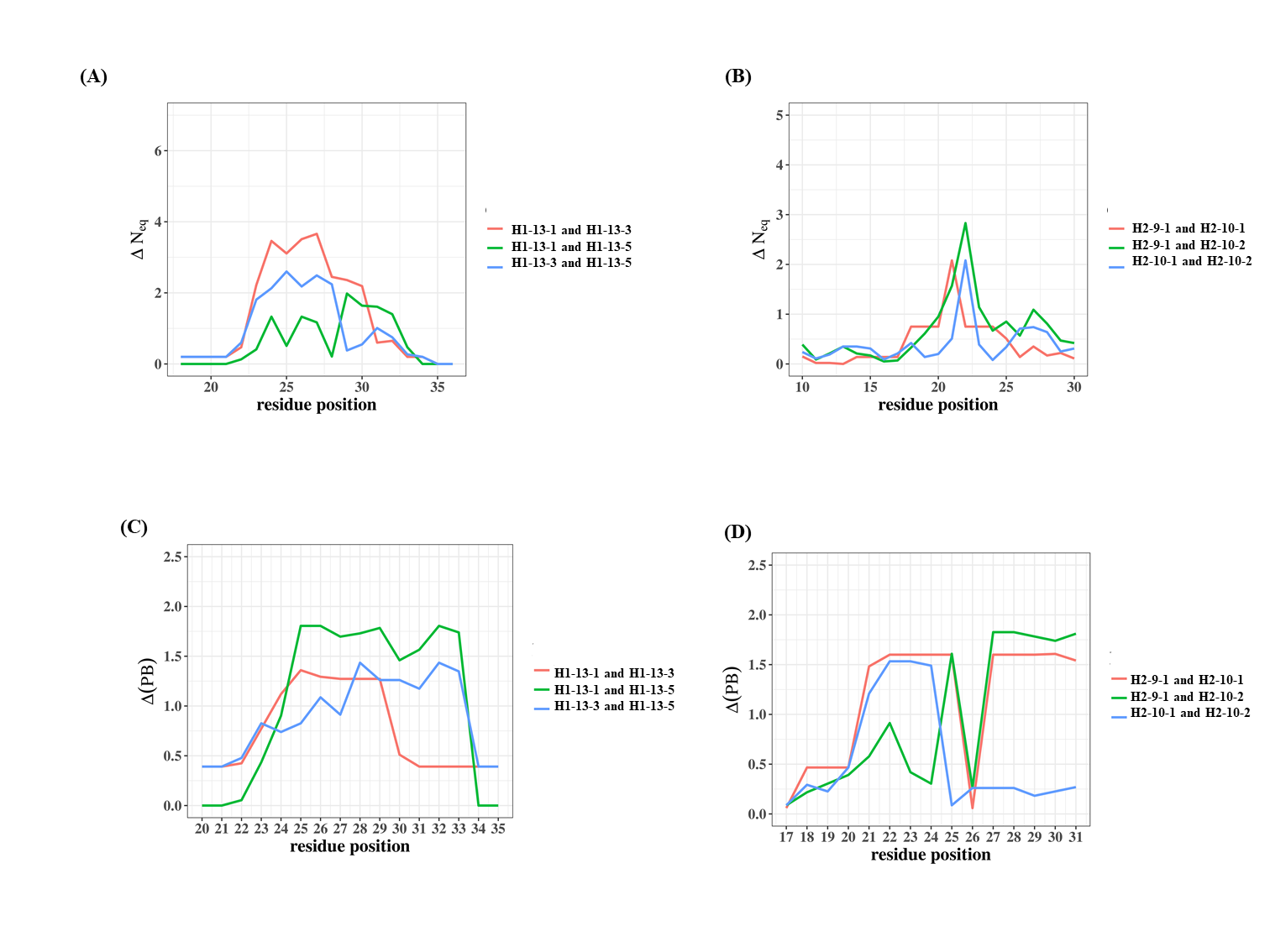

Supplement: Figure S12 — ΔN eq is shown in (A) between CDR1 clusters and (B) between CDR2 clusters and ΔPB is shown in (C) between CDR1 clusters (D) between CDR2 clusters. Legends are provided at the left of each plot. ΔN eq emphasizes a difference between H1-13-1 and H1-13-5 around a value of 1, while between H1-13-1 and H1-13-3 it is above 3. For H2-9-1, H2-10-1 and H2-10-2, profiles are very different, often with ΔN eq lower than 1 with a common peak around position 23. [file peerj-08-8408-s013.tif]

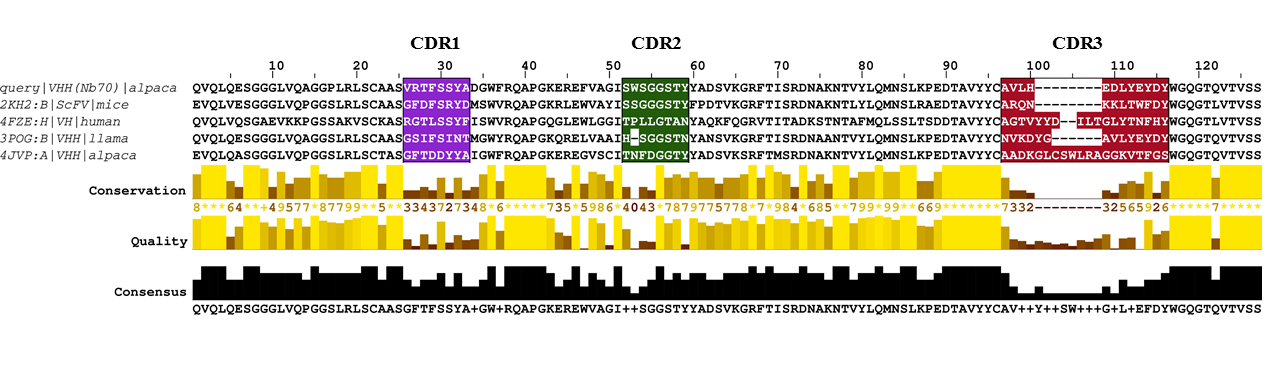

Supplement: Figure S13 — CDRs 1, 2 and 3 are demarcated according to the IMGT numbering system and coloured in pink, green and red respectively. Below the sequence alignment are the metrics conservation, quality and consensus of the alignment. [file peerj-08-8408-s014.tif]

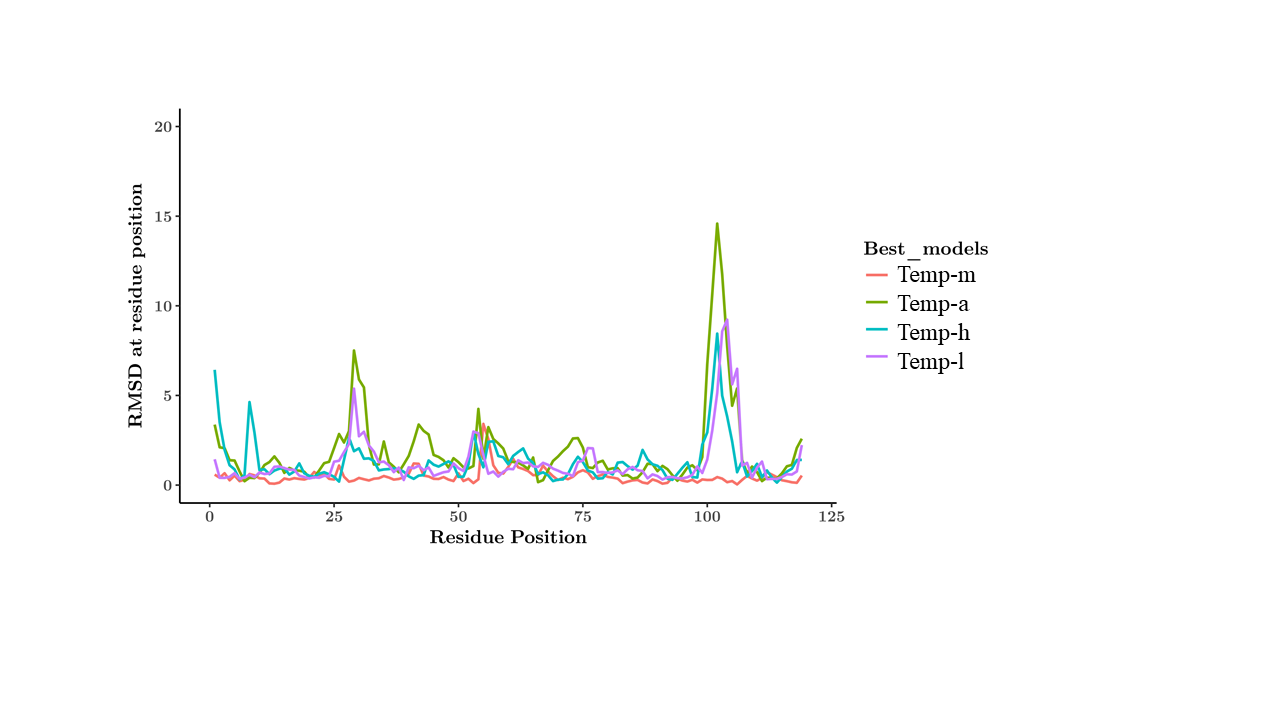

Supplement: Figure S14 — RMSD between the best structural model of the multiple template scenario and best structural models from individual template scenarios. X-axis represents residue position and Y-axis represents RMSD. [file peerj-08-8408-s015.tif]

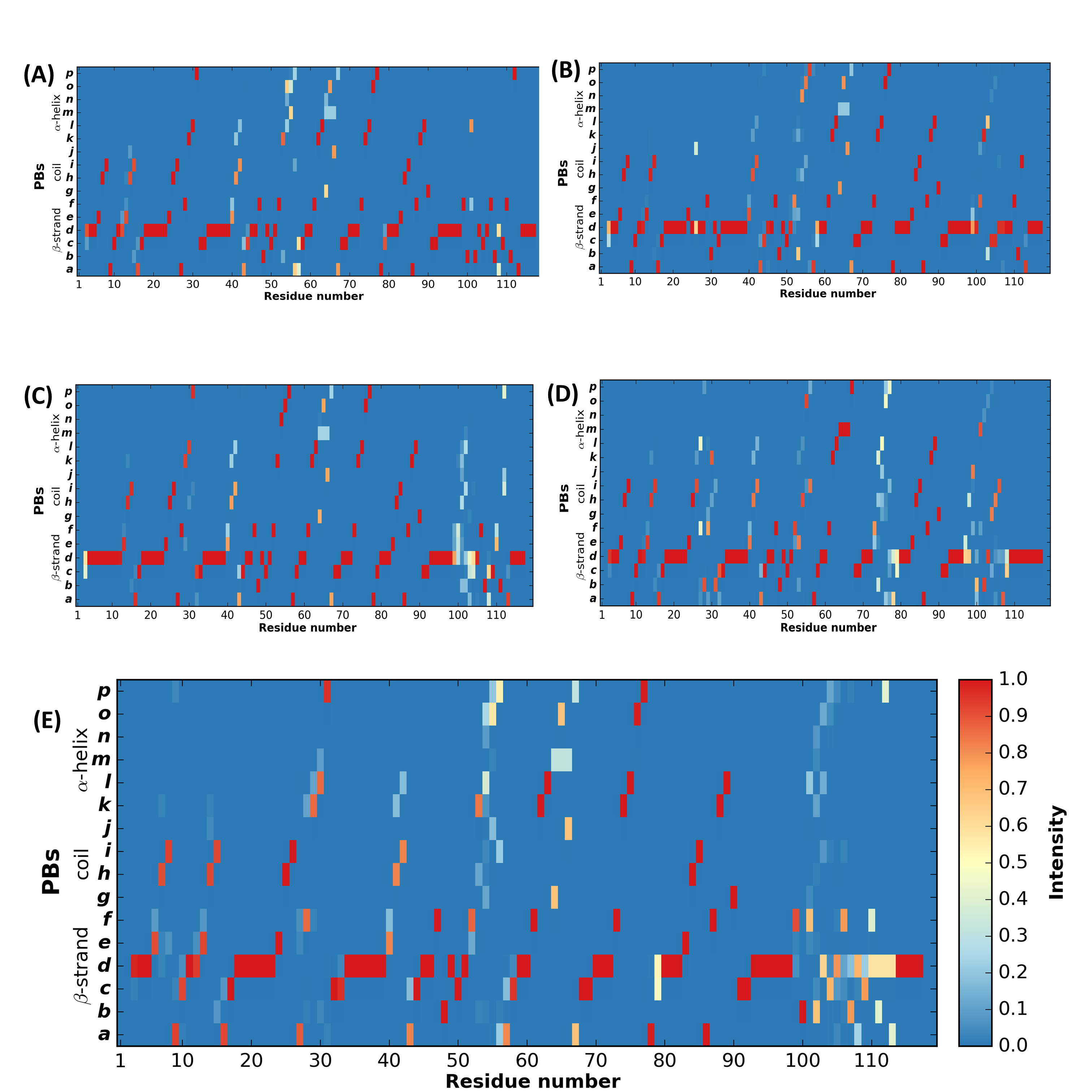

Supplement: Figure S15 — PB map of models constructed (A) using temp-m (PDB ID 2KH2), (B) temp-l (PDB ID 3P0G), (C) temp-a (PDB ID 4JVP) (D) temp-h 4FZE and (E) using all the four templates. The intensity ranges from 0 to 1, colour coded from blue to red. The X-axis on each map represents the residue position; on the Y-axis PBs are shown along with a rough secondary structure association for interpretation. [file peerj-08-8408-s016.tif]

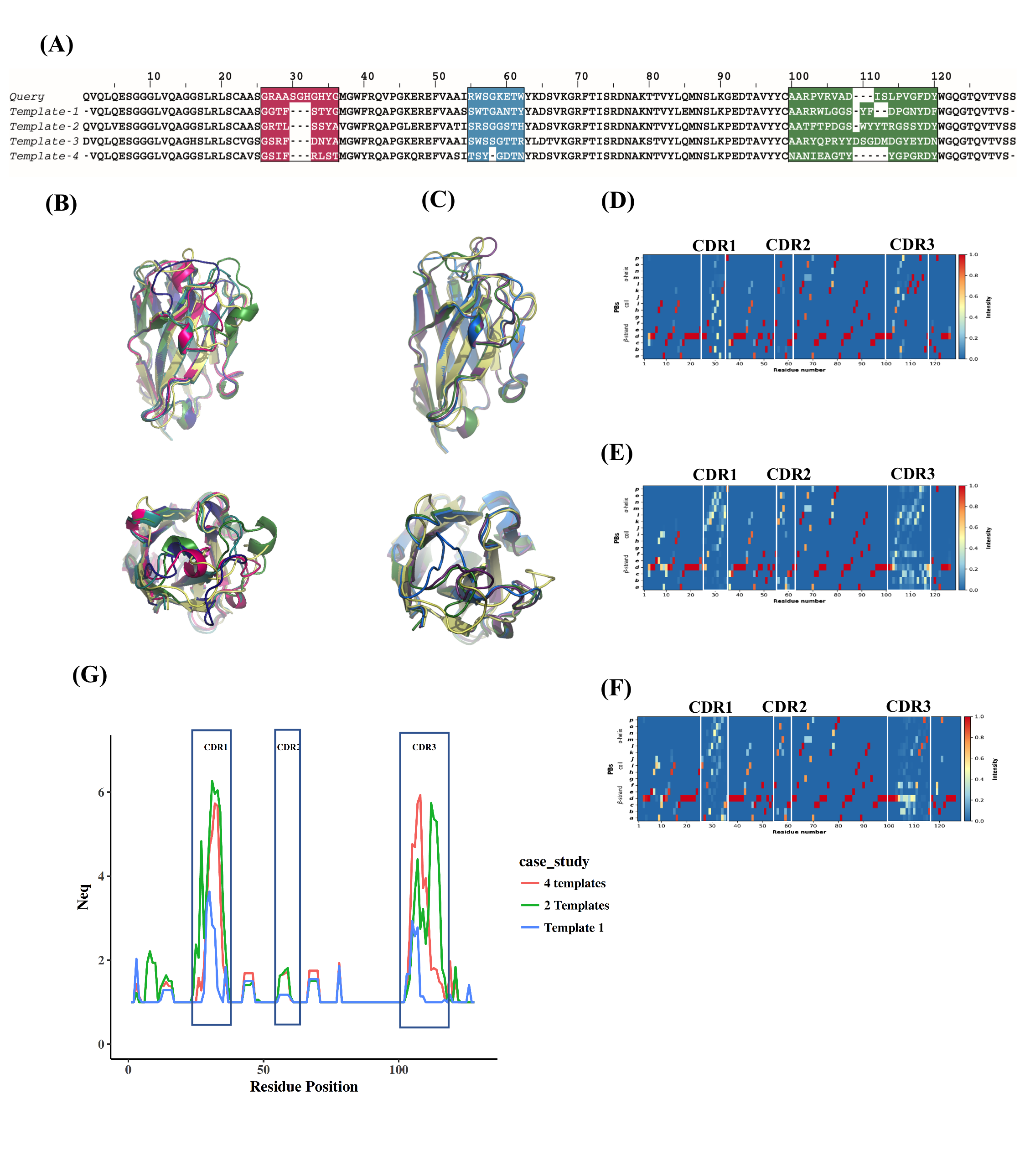

Supplement: Figure S16 — (A) Multiple sequence alignment of query V HH with the selected templates. (B) Structural superimposition of query (yellow), Template-1 (pink), Template-2 (turquoise), Template-3 (green), Template-4 (blue). (C) Structural superimposition of best models in each case with the query (yellow), best model using Template-1 (violet), best model using Templates 1 and 4 (green), using all templates (blue). (D) PB frequency map of structural models generated with Template-1. (E) PB frequency map of structural models generated with Templates 1 and 4, (F) PB frequency map of modelling scenario with four templates, (G) PB entropy at each position for each modelling scenario. [file peerj-08-8408-s017.tif]
