# Supplementary material for: Discrete analysis of camelid variable domains: sequences, structures, and in-silico structure prediction
Source: PeerJ. 2020 Mar 6;8:e8408. doi: 10.7717/peerj.8408 (PMC7061911; doi:10.7717/peerj.8408)
Supplement: Table S1 — The table provides (A) the sequence identitypercentage between structural templates and query, (B) for each FRs and CDRs (in brackets) it provides the similarity values (the best values are in bold and lowest in italics) and (C) the RMSD between structural templates. [file peerj-08-8408-s018.docx]

**(A)**

| Sequence identity (SI %) | Temp-m | Temp-l | Temp-a | Temp-h |
| --- | --- | --- | --- | --- |
| Temp-m (PDBID 2KH2) | -- |  |  |  |
| Temp-l (PDBID 3P0G) | 67.2 | -- |  |  |
| Temp-a (PDBID 4JVP) | 64.0 | 61.1 | -- |  |
| Temp-h (PDBID 4FZE) | *45.6* | *38.8* | *37.0* | -- |
| Query (Nb70) | **72.3** | **76.4** | **70.6** | *45.3* |

**(B)**

| SI (%) | FR1 | CDR1 | FR2 | CDR2 | FR3 | CDR3 | FR4 |
| --- | --- | --- | --- | --- | --- | --- | --- |
| Temp-m | 84 (84) | 37 (37) | 61 (66) | **75** (**75**) | 83 (94) | 33 (41) | *90* (*90*) |
| Temp-l | **96** (**96**) | *25* (*25*) | 72 (83) | 71 (71) | **94** (94) | **58** (**58**) | **100** (**100**) |
| Temp-a | 80 (80) | 37 (37) | **77** (77) | 37 (50) | **94** (**97**) | *8* (*25*) | **100** (**100**) |
| Temp-h | *44* (60) | **50** (**50**) | *50* (*66*) | *12* (*37*) | *51* (*64*) | 16 (25) | *90* (*90*) |

| RMSD (Å) | Temp-m | Temp-l | Temp-a | Temp-h |
| --- | --- | --- | --- | --- |
| Temp-m | -- |  |  |  |
| Temp-l | 2.4 | -- |  |  |
| Temp-a | 1.9 | 3.7 | -- |  |
| Temp-h | 2.0 | 2.2 | **4.5** | -- |

**(C)**
