# Supplementary material for: Discrete analysis of camelid variable domains: sequences, structures, and in-silico structure prediction
Source: PeerJ. 2020 Mar 6;8:e8408. doi: 10.7717/peerj.8408 (PMC7061911; doi:10.7717/peerj.8408)
Supplement: Table S2 [file peerj-08-8408-s019.docx]

| **RMSD (Å)** | **Temp-m vs Temp-l** | **Temp-m vs Temp-a** | **Temp-m vs Temp-h** | **Temp-l vs Temp-a** | **Temp-l vs Temp-h** | **Temp-a vs Temp-h** |
| --- | --- | --- | --- | --- | --- | --- |
| FR1 | 0.73 | 0.82 | 0.82 | 0.88 | **1.54** | **1.58** |
| CDR1 | 2.23 | 2.0 | 2.0 | 1.73 | 2.27 | 3.1 |
| FR2 | 0.43 | 0.77 | 0.77 | 0.76 | 0.79 | **0.97** |
| CDR2 | 2.06 | 1.63 | 2.23 | 0.81 | 0.1, 0.26 | 0.8 |
| FR3 | 0.86 | 0.50, 0.50 | 0.50, 0.50 | 1.02, 0.48 | **1.31** | **1.03, 0.87** |
| CDR3 | 0.18, 1.94 | 1.26, 0.014 | 1.26, 0.91 | 2.86, 3.07 | 2.14, 1.26 | 0.08, 2.38, 3.01 |
| FR4 | 0.88 | 0.47 | 0.47 | 0.81 | **0.98** | 0.79 |
