# Supplementary material for: Discrete analysis of camelid variable domains: sequences, structures, and in-silico structure prediction
Source: PeerJ. 2020 Mar 6;8:e8408. doi: 10.7717/peerj.8408 (PMC7061911; doi:10.7717/peerj.8408)
Supplement: Table S3 — (A) type 1 and (B) type 2. [file peerj-08-8408-s020.docx]

**A)**

| Sl no | PDB ID: CHAIN ID | Conserved Disulphide bridge  Dihedral angle pattern | Additional Disulphide bridge  Dihedral angle pattern |
| --- | --- | --- | --- |
| 1 | 1F2X:K | **+ + - - +** | **+ + + + -** |
| 2 | 1JTO:A | **- + - - +** | **- - - - -** |
| 3 | 1KXV:C | **- + - - +** | **- - - - -** |
| 4 | 1MEL:A | **- + - - -** | **- - - - -** |
| 5 | 1RI8:A | **- + - - +** | **- - - - -** |
| 6 | 1RJC:A | **- - - - +** | **- - - - -** |
| 7 | 1XFP:A | **+ - - + +** | **- - - - -** |
| 8 | 1YC8:A | **+ + + + +** | **- - + - -** |
| 9 | 1ZV5:A | **+ + - + +** | **+ + + + -** |
| 10 | 2X6M:A | **+ + - + -** | **+ + + - -** |
| 11 | 4KDT:B | **- + - - -** | **+ - - - -** |
| 12 | 4W6Y:B | **+ + - - +** | **- - - - -** |

**B)**

| Sl no | PDB ID: CHAIN ID | Conserved Disulphide bridge  Dihedral angle pattern | Additional Disulphide bridge  Dihedral angle pattern |
| --- | --- | --- | --- |
| 1 | 1KXT:B | **+ + - + -** | **+ + + - -** |
| 2 | 4C58:B | **+ + - - +** | **+ + + + -** |
| 3 | 4C59:B | **- + - - +** | **- - - - -** |
| 4 | 4GRW:F | **- + - - +** | **- - - - -** |
| 5 | 4JVP:A | **- + - - -** | **- - - - -** |
| 6 | 4LAJ:H | **- + - - +** | **- - - - -** |
| 7 | 4QGY:A | **- - - - +** | **- - - - -** |
| 8 | 4W6W:B | **+ - - + +** | **- - - - -** |
| 9 | 4Y7M:A | **+ + + + +** | **- - + - -** |
| 10 | 4Y8D:C | **+ + - + +** | **+ + + + -** |
| 11 | 4YGA:B | **- + - - -** | **+ - - - -** |
| 12 | 5HGG:T | **+ + - - +** | **- - - - -** |
| 13 | 5IVN:A | **+ + + + +** | **- - - - -** |
