# Supplementary material for: Discrete analysis of camelid variable domains: sequences, structures, and in-silico structure prediction
Source: PeerJ. 2020 Mar 6;8:e8408. doi: 10.7717/peerj.8408 (PMC7061911; doi:10.7717/peerj.8408)
Supplement: Data S1 [file peerj-08-8408-s021.docx]

***Analysis of local conformations of CDR2 of V_H_H****.* The CDR2 region is defined from residue positions 56 to 68. In the 29 clusters of CDR-H2, 16 clusters have V_H_H structures, of these 13 are sparsely populated (< 10 structures). The remaining three clusters were analysed as described for previously. Figure 4H shows *N_eq_* profiles and emphasize conformational diversity compared to CDR1. The first cluster, H2-9-1 (see Figure 4D) is highly directed with near no variation of PBs for its 28 structures and the PB series *dfbdcdfkopacddfklg*. *N_eq_* values are higher for the two other analysed clusters at positions 56 to 62 (see Figure 4H). Nonetheless, cluster H2-10-1 (see Figure 4E) has 14 structures with PB series *dfbdcdfk****l****opacddfkl* and interestingly the second common PB motif *dfbdcdfk****n****opacddfkl* seen in 8 structures, which differ at only one PB position (underlined). Cluster H2-10-2 (see Figure 4F) also exhibits similar behaviour with the two most common PB series *dfbdcdfko****p****accddfkl* seen in 18 and *dfbdcdfko****m****accddfkl* seen in 8 structures differing at one position (underlined). The flanking regions in all these clusters are identical at the N-termini of the loop. The C-termini although has a classic helical entry PB motif *fkl*. The PB structural word *dfkop* seen in H2-9-1, H2-10-2 is rarely observed, as it is also the case with the structural word seen *nopac* seen in second common motif of cluster H2-10-1.
