# Supplementary material for: Discrete analysis of camelid variable domains: sequences, structures, and in-silico structure prediction
Source: PeerJ. 2020 Mar 6;8:e8408. doi: 10.7717/peerj.8408 (PMC7061911; doi:10.7717/peerj.8408)
Supplement: Supplemental Information 1 [file peerj-08-8408-s022.docx]

***Supplementary Results:***

***Detailed description of VHH structure prediction studies:***

*1. Anti- DARC*. In this study V_H_H raised against human Duffy Antigen / Receptor for Chemokines (DARC) had been obtained from immunised dromedary. DARC is the entry point of malarial parasite, *Plasmodium vivax*, into red blood cells. This is a first study attempting modelling of V_H_H where the authors modelled an anti-DARC V_H_H (named CA52) using two templates 1OP9(1) and 1JTO(2) selected using PSI-BLAST against PDB. The query is 124 amino acids in length and has an extra disulphide bond between CDR1 and CDR3. The additional CDR1-CDR3 cysteine bond was constrained as found in the second template later template Two hundred models for each template were generated using Modeller. Comparison of the models was done by superimposition (the Cα backbone using ProFit software); the model with mean RMSD, i.e. the barycentre of the models was selected as a plausible near-native structural model. This unorthodox approach was chosen, as the DOPE score was non-discriminative due to the extremely high structural similarity of the models. The details of the approach are provided in(3), and underlined the importance to analyse specifically each regions of V_H_H sequence as it can change entirely the local protein conformations. This paper also underlines the importance of electrostatics in the binding of this V_H_H.

*2. Anti-glycoprotein, Anti-lysozyme and Anti-prostate specific antigen.* Three different V_H_Hs were used in the study anti-glycoprotein (cAbAn33), anti-lysozyme (cAbLys3), and anti-prostate specific antigen (cAbPSA-N7) to explore the contribution of an extra disulphide bond (between CDR1 and CDR3) present in camels and llamas to the stability of these proteins. Two of these proteins cAbAn33 and cAbLys3 have their crystal structures are already known. These were used for creating models of mutants replacing the extra cysteines CDR1 and CDR3. ESyPred3D(4), i.e. a methodology based on Modeller and Robetta(5) have been used to generate the mutant models. The authors mention that the models from ESyPred correspond with those generated by Robetta (data has not been shown). ESyPred3D has two schemes to select best models, i.e. AL0 (an alignment specific score) of the template and the query and GDT_TS of the templates. (6). Molecular modelling in this study has been useful in understanding the structural conservation of mutants in terms of fold. The models of the mutants predicted had very fewer differences compared to their parent VHH structures.

*3. Specific approach for V_H_H structure prediction.* Rosetta(7) is the most successful *de novo* approaches along with I-Tasser(8). A specific development called RosettaAntibody (9) which Gray’s group, the first to put forward a specialised modelling protocol for V_H_H. They have modified their Rosetta Antibody suite to model single chain V_H_H. They used 27 PDB structures in their study, splitting them into test and training set. Analysis did not include alpaca V_H_H. The templates for FR regions are selected using BLAST against antibody databank containing structures from PDB and for CDRs the templates were chosen from BLAST bit scores. Once the FRs are modelled, the CDRs is grafted onto the FRs by optimal superposition of the backbone atoms of two overlapping residues at each end of the loop. This study underlines the difficulty in V_H_H modelling and specifically the fact that an approach for IgG is not optimal for V_H_H. The specific script for V_H_H are not available anymore in the newer version of RosettaAntibody for classical antibodies, but it is possible to model V_H_H by submitting a dummy light chain in the protocol and then deleting it from the models once they are modelled.(10)

*4. Anti- Phospholipase A2.* Phospholipase A2 (PLA2) is an enzyme present in snake venom, which digests cell membrane lipids. The number of snakebite cases especially due to monocled cobra (*Naja kaouthia*) in Thailand is on rise. An antibody against PLA2 has interest, especially a camelid V_H_H, due to its thermostable properties. Humanised dromedary V_H_H phage library, which was prepared in a previous study by the same group, was used. Two V_H_H clones from PLA2 bio panning (P3-1, P3-3) were selected for further studies of modelling and docking. Both V_H_H clones have all the signatures of V_H_H along with P3-3 having an extra CDR1-CDR3 disulphide bond. The templates selected using BLAST against PDB, which were 1VHP (11) (camelised V_H_) for P3-1 and 1MVF (12)(V_H_H) for P3-3. The structures have been modelled by Discovery studio that uses Modeller. The steric hindrance in the models was assessed using Ramachandran plot. The best models were taken for docking with PLA2 using ZDOCK. (13) The docking experiments revealed the models bind to the Ca^2+^ binding site, the active site and the phospholipid-binding site on the PLA2. These findings are yet to be experimentally verified.

*5. Anti-NS5B.*Non-structural protein 5B is a RNA dependent RNA polymerase (RdRp) in Hepatitis C Virus (HCV). It plays an important role in HCV infection cycle. In this study V_H_ / V_H_H against Non-structural protein 5B (NS5B) from dromedary expressed using a phage library and selected using bio panning were modelled using homology modelling (algorithm not explicitly mentioned). The templates 1VHP(11), 1F2X (14) were chosen to model two V_H_H clones, V_H_H6 and V_H_H 24 that were selected based on sequence identity in BLAST searches. The models were evaluated using ProCheck and showed 0% of residues outside the allowed region. Further the models were docked on to the NS5B using ZDOCK and RDOCK modules in Discovery studio (15). Analysis of the docked complexes suggested that the V_H_H models bind to the catalytic groove of the NS5B RdRp preventing the template from binding.

*6. Anti-HEWL.* An interesting study about altering the affinity of V_H_H with modifying paratope instead of epitope was carried out in a previous study by the same group. In this study, is an extension of the same about the effect of point mutations in CDR1 and CDR2 of various residues to tyrosine and serine in V_H_H bound to Hen egg white lysozyme (HEWL) with predetermined structure PDBID: 1RI8 (16) were carried out using MOE from chemical computing group. Molecular mechanics in MOE suite was used to obtain the local minima of the complex followed by molecular dynamics of the complex to investigate the global minima of the modelled complex.(17) Molecular modelling in this case has helped to reduce the experimental time required in understanding the biophysical properties of the mutants. As the mutants were modelled as a complex with HEWL same as the parent, their effects through CDR2 binding were explained using the above-mentioned *in silico* approaches.

*7. Anti-NS3-C.*  The C-ter of non-structural protein S3 of Hepatitis C Virus (HCV) has helicase-NTPase activity. Its activity is crucial during replication; it helps in separation of the positive and negative sense RNA. In this study, humanised V_H_/V_H_H against the HCV viral Helicase NS3-C were generated and assessed for their binding regions. Three V_H_/V_H_H were found to be attractive targets, namely V_H_6, V_H_H9 and V_H_59. Templates 1OHQ (18), 1XFP (19) and 3BN9 (20)were chosen respectively based on sequence identity. The precise protocol of homology modelling was not detailed; the models were assessed using Ramachandran plot for disallowed backbone conformations(21). The models were further docked on NS3-C using ZDOCK, to validate its epitope regions of the suggested through experiments.

*8. Anti-Urease.*  Urease C of *Helicobacter pylori* has an important role to play in bacterial colonisation of the gastric mucosa. Loss of its activity has been speculated to arrest *H. pylori* colonisation, thus making it a target for therapeutic intervention. This study involves characterisation of anti-UreC (Urease C) and its structure prediction by fold prediction software Phyre2(22). A high affinity mutant form called as HMR23 was also generated to understand the binding properties with respects the parent anti-UreC. The template selection protocols and model validation statistics are not mentioned.(23). Modelling of parent and mutant VHH was essential to understand the structural conservation in terms of fold in the later.

*9. Anti-TNFR1α (Tumour necrosis factor receptor 1 α).* Tumour necrosis factor (TNF) is a cytokine, which has an important role to play in immune regulation. It functions through its receptors TNFR1 and TNFR2. TNFR1 activates apoptosis pathways and TNFR2 plays a role in tissue homeostasis. This study was aiming to inhibit TNFR1 α using camelid V_H_H. Of the many V_H_H purified two of them (Nb 70 and Nb 96) were chosen for docking studies based on experimental observations. Both were modelled using Modeller using multiple templates belonging to 4 different structures (namely PDB IDs 4FZE(24), 4JVP (25), 2KH2(26), 3P0G (27)). The models generated were further validated by RAMPAGE (28)software and the best models ranked were used for docking. The docking studies have further helped in identifying that there is a difference in mode of binding between the two nanobodies, Nb 70 binds in the catalytic domain of hTNFR1 whereas Nb 96 does not. The authors have not mentioned the algorithm for template selection in their protocols. We have further analysed this particular case of Nanobody modelling using the same templates used by the authors in both multi template method and individual templates. We have presented it as a case study in the later sections. (29)

*10. Anti-VEGFR2 (Vascular endothelial growth factor receptor 2).* Vascular endothelial growth factor (VEGF) is very crucial for angiogenesis. It has two receptors VEGFR1 and VEGFR2. Though VEGFR1 has more affinity towards its ligand VEGF, VEGFR2 mediates VEGF signalling predominantly. This study attempts to inhibit VEGF signalling through VEGFR2 in cancer cells (HUVECs). In this study three V_H_H clones were generated and selected against the VEGF-RBD (VEGF Receptor Binding Domain). To model these clones namely VEvhh1, VEvhh2 and VEvhh3, templates were selected from BLAST search against PDB namely 1OP9 (1), 1MVF (12) and 2X6M (30). These were modelled using Modeller and successively the CDR loops were refined using the Loop refinement protocol by Modeller. Further the models were validated using ProCheck. The best model of each V_H_H was refined using a detailed MD protocol, after which they were proceeded for docking. (31) After a detailed protocol of docking involving three rounds using HADDOCK, the accessible surface difference (ΔASA) between the VHH (each of VEvhh1, VEvhh2 and VEvhh3) and VEGF-RBD complex and isolated VEGF-RBD was calculated. Amongst the three nanobodies, VEvhh1 showed maximum coverage of functional regions of VEGF, which binds to VEGFR2.

*11. Anti-NS3/4A.* Hepatitis C virus has 6 non-structural proteins (NS), NS2, NS3, NS4A, NS4B, NS5A and NS5B. Of them the fusion protein NS3 and NS4 form a serine protease which cleaves the linker between NS5A and NS5B. NS5B is in turn highly implicated in the virus life cycle. Hence arresting the activity of before NS5B can prove to be critical. In this study three V_H_H clones were generated against the fusion HCV Protease, NS3/4A. I-TASSER webserver was used to model three V_H_H sequences selected from experiments named V_H_H24, V_H_H28 and V_H_H41. Modeller was used to generate NS3/4A models. No specific details about templates or primary sequence information is furnished in the paper (32) The best models in each case were refined using ModRefiner and fragment guided molecular dynamics. Later the models were docked to the target. Docking analyses revealed that CDR2 and CDR3 in all the three VHH bind to the NS3/4A catalytic triad residues. In case of models of V_H_H28 and V_H_H41, residues from framework regions were also implicated in interactions with NS3/4A in the docking analysis.

*12. Anti-CDT. Clostridium difficile* Toxin (CDT) produced by *Clostridium diffcile* bacterium, is a potent toxin responsible for antibiotic-associated diarrhoea. These toxins belong to C2 class of toxins, which means these have two subunits (CDTa and CDTb). CDTa subunit is responsible for ADP-ribosylation of actin and CDTb is responsible for forming a toxin-pore complex through which the CDTa subunit is internalised. This study attempts to inhibit the function of CDTa subunit using V_H_H from lama. Three V_H_H clones selected from experimental studies (V_H_H 1+8, V_H_H 1-14, V_H_H 1+18) were modelled using BioLuminate package from Schrödinger. The modelling protocol has two stages, first stage of modelling Framework Regions (FRs) with templates (selected using sequence identity) obtained from a curated antibody database inside the same tool. Next, CDRs are grafted from another custom database of CDR loops in BioLuminate, based on structural clustering, sequence similarity and stem residue geometry matching. Two of the clones had an extra disulphide bond which were preserved in the models. Further the models were proceeding for docking to understand their precise mode of interaction. (33) The models were docked on to CDTa using PIPER module in Discovery studio, which further aided in understanding the precise mode of interaction between V_H_H and CDTa. All three V_H_H bound to the NAD^+^ binding cavity in CDTa, two of the V_H_H (1-14 and 1+18) were known to block each other, whereas V_H_H 1+8 bound CDTa at an independent binding site. This study has also reported interactions in framework regions of the V_H_H used.

*13*. *Anti-PrA (Protein A).* Protein A (PrA) from staphylococcus has interesting property of binding to the F_C_ and sometimes F_ab_ regions of antibodies, which makes it an attractive tool for in vitro isolation of antibodies. This study attempts to understand the binding of PrA to V_H_H. The authors designed V_H_H proteins with minimal CDRs to remove their contribution in binding to PrA. I-TASSER web server was used to model the structure of LaP-1(Llama antibody against Protein A) nanobody and its mutants. The information of templates chosen and the quality of the models were not particularly discussed in detail. The best models were chosen based on the c-score of the I-TASSER(34). Authors underlined that the models provided insights into the structural stability of the mutants and the parent V_H_H.

*14. Anti-BMP4.* Bone morphogenic factor 4 (BMP4) protein is a transcription factor for many genes. Some are involved in various cancers, thus making BMP4 an interesting drug target. In this study three V_H_Hs clones (E7, C8 and C4) from experimental studies against BMP4 were selected based on different biophysical and biochemical properties. Using Modeller, C4 was modelled using templates PDB ID 4BSE (35) and 1SJX (36) template was used for modelling E7 and C8. These templates were chosen based on their sequence identity. There are no specific details of model validation before proceeding for docking with BMP4.(37) HADDOCK server was used for docking of all three V_H_Hs to BMP4. Analyses of docking poses revealed that the differences in modes of binding between the three nanobodies, V_H_H C4 and V_H_H C8 bind to wrist epitope of BMP4 at non-overlapping regions, whereas V_H_H E8 bound to the knuckle epitope region of BMP4. These epitopes are known in binding to BMP receptor 1a. The above-mentioned *in silico* analyses suggest that the mode of action of these nanobodies is to occlude the BMPR1a binding site, thereby inhibiting the downstream process.

15. *Anti- BthTX-I and BthTX-II. Bothrops jararacussu* belonging to class of Viperidea family, is responsible for most mortality of snake bites Central and South America. The main component of snake venom is phospholipases, which dissolve the cell membranes. This study is aimed to make anti-venom against two phopholipase 2-like (PLA2) proteins in the venom from *B. jararacussu* namely, Bothropstoxin-I (BthTX-I), a Lys-49 PLA_2_-like and Bothropstoxin-II (BthTX-II), an enzymatically active Asp-49 PLA_2_. In this study four llama V_H_H clones against BthTX-I and II were selected based on experimental studies. These V_H_H clones ([KF498607](https://www.ncbi.nlm.nih.gov/nuccore/KF498607), [KF498608](https://www.ncbi.nlm.nih.gov/nuccore/KF498608), [KC329715](https://www.ncbi.nlm.nih.gov/nuccore/KC329715) and [KC329718](https://www.ncbi.nlm.nih.gov/nuccore/KC329718)) were modelled using templates selected from Protein BLAST against PDB as 4KRP(38) ,4DKA (39), 3EZJ (39) and 4KRP(38) respectively. All the templates had a resolution under 3.0 Å. The templates and query sequences were aligned using ClustalW(40) and modelled using Modeller. A total of 1000 models were generated and the best were selected using lowest DOPE scores. Structural validation of the best models was done using ProCheck(41). The best model in each case had a RMSD of < 1 Å with respective templates.(42) The models of KF498607 and KC329715 have shown binding to the C-terminus of the toxins, which is the site for its membrane docking. These both V_H_H seem to bind to enzymatic cleft of the BthTX-II inhibiting Ca^2+^ binding required for its activity. The other V_H_H KF498608 also showed similar interaction profile to that of KF498607, but KC329715 did not show any “good” interaction with the toxins in the clusters.

16 *Anti-histone*. Histones are a part of nucleosome complex around which DNA is wrapped inside the nucleus. Some post-translational modifications of histones are indicators of gene expression in that particular region of chromosome. This feature of histones can be exploited to understanding chromatin dynamics by having exogenous protein that bind to them. In this study VHH raised against the H2A-H2B complex in the chromatin were named as chromatibody. Experimentally tested chromatibody was modelled using Modeller from Discovery Studio. Template selection protocol was not clearly mentioned in the paper. The best models were ranked based on molpdf and DOPE scores. The best of the models from the ranking were further validated using profile-3D scores for stereo chemical clashes. (43) Molecular modelling in this study has been helpful in suggesting the appropriate placement chromatin-binding motif in the β- hairpin region of the CDR3 into the H2A-H2B cavity.

*17. Anti-MMP8*. Matrix Metalloproteinases (MMP) are proteases that degrade the extra cellular matrix and have important role cell migration during organogenesis and metastasis. High expression of MMP8 is seen many pathologies like arthritis, cancer, neuroinflammation to name a few (44). One such study was done using a potent V_H_H(Nb14) against MMP8 (both human and mouse isoforms). Multiple templates were used to model the query V_H_H(Nb_14_) using Modeller with templates 4LAJ(45), 3EZJ(46), 3TPK(47) and 4M3J(48). The template selection protocol was not explicitly mentioned. Further the models generated were validated using RAMPAGE software(28) and the best models were used for docking using ClusPro. The docking analysis revealed that the Nb_14_ does not bind to the active site of MMP8, but to three other sites on the enzyme. The inhibition of MMP8 by Nb_14_ is then explained to be through steric hinderance or by induction of other conformational changes. (49)

*18. Anti-bap. (Biofilm associated protein). Acinetobacter baumannii* is a multi-drug resistant bacterial species responsible for many hospital-derived infections. Its ability to form bio-films helps it survive hospital conditions. Thus, preventing the formation of bio-film can contribute to the reduction of infection. In this study, a single V_H_H clone against Bap (Biofilm associated protein) proved efficient in a previous study by the same group. The authors have used several approaches to model this protein. The Protein Structure Prediction Server (PS^2^V^2^)(50) was used for selecting the templates and aligning them with the query. It also uses Modeller to model the query given an alignment. They have also modelled the anti-Bap using fold prediction server Phyre2. Another attempt to model the anti-Bap clone was done using LOMETS. All the templates belong to the camelid V_H_Hs. A total of ten models were generated with ten different templates. Model evaluations have been done using ProSA (51,52). The model generated by PS2V2, which uses Modeller, has better percentage of residues (98%) in the favourable region of Ramachandran plot than the other models. These models were further refined using Modrefiner for further studies. (53) The docking analysis by ZDOCK suggested that anti-bap V_H_H binds to Bap at its N-terminus (no specific information is provided in the paper).

*19.* *ABodyBuilder: Automated antibody structure prediction with data–driven accuracy estimation*: Antibody specific modelling approaches have an advantage of robust/advanced loop modelling protocols incorporated in them compared to other generic protein modelling algorithms. The most recent in this class of antibody-specific modelling approaches is ABodyBuilder, generic antibody modelling server that can be used for V_H_H also. Templates are selected using sequence identity of the FR regions as a criterion against SAbDab (54). It then uses Modeller for modelling FR regions. Next, CDRs are modelled using FREAD, an algorithm developed by their group for modelling loops using database search. FREAD basically searches against the CDR database created for each CDR (six in total). The SAbDab is the first database to have specific text search for querying V_H_H, however the text search also lists modified V_H_/V_L_ which exist as single domain antibodies.(55,54)

*20. Anti-lysozyme.* The demand for V_H_H in therapeutics and diagnostics is on rise. Like most proteins, bulk production in-vitro of V_H_H is influenced by various biophysical properties. A comprehensive understanding of the degree of the influence of amino acid substitutions and CDRs on these above-mentioned factors through Molecular dynamics and docking is the main goal of this study. Three V_H_H proteins with known structures namely NbHuL6, cAbLys and cAbCII10 were used in the study. The FRs of NbHuL6 were modelled with those of PDBID: 3EBA(56) and FRs of cAbLys and cAbCII10 were modelled with PDBID: 1JTP(2) and PDBID 1ZMY(57). The reasons for selecting these templates are specific to the study. The loops were modelled using templates from PDBID 3DWT(56) and 1ZMY(57). These chimeric VHH were manually built after superposition. The amino acid mutants in the FR2 region were generated using Swiss Pdb-viewer. The mutants and chimeras were assessed for thermos-stability using molecular dynamics and colloidal interactions using docking to understand the interplay of the biophysical factors on the yield of respective mutants or chimeras (58).

*21. Anti-CyaA-Hly.* Two V_H_(V_H_5,V_H_18) and two V_H_H (V_H_H2,V_H_H37) clones were screened from the phage display against the pertussis adenylate cyclase-hemolysin toxin and the repeat in toxin (CyaA-RTX protein) sub domains. V_H_5 and V_H_18 were modelled using 4O9H(24) and 2KH2(26) and the V_H_H clones V_H_H 2 and V_H_H 37 were modelled using 1F2K(24) and 4HEP(59) respectively as templates. 3D models of these proteins were built using RaptorX (60,61)with further assistance from loop modelling from FALC (62)loop modelling server. The best models were validated using various algorithms on saves server and the best models were energy minimised using Gromacs.(63) The V_H_H models were docked on the target CyaA-RTX domains to find understand the mode of interaction between them. All the nanobodies were found to interact with the target especially in the linker region between the two domains CyaA and RTX.

References:

1. Dumoulin M, Last AM, Desmyter A, Decanniere K, Canet D, Larsson G, Spencer A, Archer DB, Sasse J, Muyldermans S, et al. A camelid antibody fragment inhibits the formation of amyloid fibrils by human lysozyme. *Nature* (2003) **424**:783–8. doi:10.1038/nature01870

2. Decanniere K, Transue TR, Desmyter A, Maes D, Muyldermans S, Wyns L. Degenerate interfaces in antigen-antibody complexes. *J Mol Biol* (2001) **313**:473–8. doi:10.1006/jmbi.2001.5075

3. Smolarek D, Hattab C, Hassanzadeh-Ghassabeh G, Cochet S, Gutiérrez C, de Brevern AG, Udomsangpetch R, Picot J, Grodecka M, Wasniowska K, et al. A recombinant dromedary antibody fragment (VHH or nanobody) directed against human Duffy antigen receptor for chemokines. *Cell Mol Life Sci* (2010) **67**:3371–87. doi:10.1007/s00018-010-0387-6

4. Lambert C, Léonard N, De Bolle X, Depiereux E. ESyPred3D: Prediction of proteins 3D structures. *Bioinformatics* (2002) doi:10.1093/bioinformatics/18.9.1250

5. Kim DE, Chivian D, Baker D. Protein structure prediction and analysis using the Robetta server. *Nucleic Acids Res* (2004) **32**:W526-31. doi:10.1093/nar/gkh468

6. Govaert J, Pellis M, Deschacht N, Vincke C, Conrath K, Muyldermans S, Saerens D. Dual beneficial effect of interloop disulfide bond for single domain antibody fragments. *J Biol Chem* (2012) **287**:1970–9. doi:10.1074/jbc.M111.242818

7. Rohl CA, Strauss CEM, Misura KMS, Baker D. Protein Structure Prediction Using Rosetta. doi:10.1016/S0076-6879(04)83004-0

8. Zhang Y, Murzin A, Bateman A, Ginalski K, Rychlewski L, Baker D, Sali A, Skolnick J, Fetrow J, Kolinski A, et al. I-TASSER server for protein 3D structure prediction. *BMC Bioinformatics* (2008) **9**:40. doi:10.1186/1471-2105-9-40

9. Sircar A, Kim ET, Gray JJ. RosettaAntibody: antibody variable region homology modeling server. *Nucleic Acids Res* (2009) **37**:W474-9. doi:10.1093/nar/gkp387

10. Sircar A, Sanni KA, Shi J, Gray JJ. Analysis and modeling of the variable region of camelid single-domain antibodies. *J Immunol* (2011) **186**:6357–67. doi:10.4049/jimmunol.1100116

11. Riechmann L. Rearrangement of the former VL interface in the solution structure of a camelised, single antibody VH domain. *J Mol Biol* (1996) **259**:957–69. doi:10.1006/jmbi.1996.0373

12. Loris R, Marianovsky I, Lah J, Laeremans T, Engelberg-Kulka H, Glaser G, Muyldermans S, Wyns L. Crystal structure of the intrinsically flexible addiction antidote MazE. *J Biol Chem* (2003) **278**:28252–7. doi:10.1074/jbc.M302336200

13. Chavanayarn C, Thanongsaksrikul J, Thueng-in K, Bangphoomi K, Sookrung N, Chaicumpa W. Humanized-Single Domain Antibodies (VH/VHH) that Bound Specifically to Naja kaouthia Phospholipase A2 and Neutralized the Enzymatic Activity. *Toxins (Basel)* (2012) **4**:554–567. doi:10.3390/toxins4070554

14. Decanniere K, Muyldermans S, Wyns L. Canonical antigen-binding loop structures in immunoglobulins: more structures, more canonical classes? *J Mol Biol* (2000) **300**:83–91. doi:10.1006/jmbi.2000.3839

15. Thueng-in K, Thanongsaksrikul J, Srimanote P, Bangphoomi K, Poungpair O, Maneewatch S, Choowongkomon K, Chaicumpa W. Cell Penetrable Humanized-VH/VHH That Inhibit RNA Dependent RNA Polymerase (NS5B) of HCV. *PLoS One* (2012) **7**:e49254. doi:10.1371/journal.pone.0049254

16. De Genst E, Silence K, Ghahroudi MA, Decanniere K, Loris R, Kinne J, Wyns L, Muyldermans S. Strong in vivo maturation compensates for structurally restricted H3 loops in antibody repertoires. *J Biol Chem* (2005) **280**:14114–21. doi:10.1074/jbc.M413011200

17. Inoue H, Suganami A, Ishida I, Tamura Y, Maeda Y. Affinity maturation of a CDR3-grafted VHH using in silico analysis and surface plasmon resonance. *J Biochem* (2013) **154**:325–332. doi:10.1093/jb/mvt058

18. Jespers L, Schon O, James LC, Veprintsev D, Winter G. Crystal structure of HEL4, a soluble, refoldable human V(H) single domain with a germ-line scaffold. *J Mol Biol* (2004) **337**:893–903. doi:10.1016/j.jmb.2004.02.013

19. De Genst E, Handelberg F, Van Meirhaeghe A, Vynck S, Loris R, Wyns L, Muyldermans S. Chemical basis for the affinity maturation of a camel single domain antibody. *J Biol Chem* (2004) **279**:53593–601. doi:10.1074/jbc.M407843200

20. Farady CJ, Egea PF, Schneider EL, Darragh MR, Craik CS. Structure of an Fab-protease complex reveals a highly specific non-canonical mechanism of inhibition. *J Mol Biol* (2008) **380**:351–60. doi:10.1016/j.jmb.2008.05.009

21. Phalaphol A, Thueng-in K, Thanongsaksrikul J, Poungpair O, Bangphoomi K, Sookrung N, Srimanote P, Chaicumpa W. Humanized-VH/VHH that inhibit HCV replication by interfering with the virus helicase activity. *J Virol Methods* (2013) **194**:289–299. doi:10.1016/j.jviromet.2013.08.032

22. Kelley LA, Mezulis S, Yates CM, Wass MN, Sternberg MJE. The Phyre2 web portal for protein modeling, prediction and analysis. *Nat Protoc* (2015) **10**:845–858. doi:10.1038/nprot.2015.053

23. Hoseinpoor R, Mousavi Gargari SL, Rasooli I, Rajabibazl M, Shahi B. Functional mutations in and characterization of VHH against Helicobacter pylori urease. *Appl Biochem Biotechnol* **172**:3079–3091. doi:10.1007/s12010-014-0750-4

24. Klarenbeek A, Blanchetot C, Schragel G, Sadi AS, Ongenae N, Hemrika W, Wijdenes J, Spinelli S, Desmyter A, Cambillau C, et al. Structure of Interleukin-6 in complex with a Camelid Fab fragment. *doi.org* doi:10.2210/pdb4o9h/pdb

25. Tarr AW, Lafaye P, Meredith L, Damier-Piolle L, Urbanowicz RA, Meola A, Jestin J-L, Brown RJP, McKeating JA, Rey FA, et al. An alpaca nanobody inhibits hepatitis C virus entry and cell-to-cell transmission. *Hepatology* (2013) **58**:932–9. doi:10.1002/hep.26430

26. Wilkinson IC, Hall CJ, Veverka V, Shi JY, Muskett FW, Stephens PE, Taylor RJ, Henry AJ, Carr MD. High resolution NMR-based model for the structure of a scFv-IL-1beta complex: potential for NMR as a key tool in therapeutic antibody design and development. *J Biol Chem* (2009) **284**:31928–35. doi:10.1074/jbc.M109.025304

27. Rasmussen SGF, Choi H-J, Fung JJ, Pardon E, Casarosa P, Chae PS, Devree BT, Rosenbaum DM, Thian FS, Kobilka TS, et al. Structure of a nanobody-stabilized active state of the β(2) adrenoceptor. *Nature* (2011) **469**:175–80. doi:10.1038/nature09648

28. Lovell SC, Davis IW, Arendall WB, de Bakker PIW, Word JM, Prisant MG, Richardson JS, Richardson DC. Structure validation by Cα geometry: ϕ,ψ and Cβ deviation. *Proteins Struct Funct Bioinforma* (2003) **50**:437–450. doi:10.1002/prot.10286

29. Steeland S, Puimège L, Vandenbroucke RE, Van Hauwermeiren F, Haustraete J, Devoogdt N, Hulpiau P, Leroux-Roels G, Laukens D, Meuleman P, et al. Generation and Characterization of Small Single Domain Antibodies Inhibiting Human Tumor Necrosis Factor Receptor 1. *J Biol Chem* (2015) **290**:4022–4037. doi:10.1074/jbc.M114.617787

30. De Genst EJ, Guilliams T, Wellens J, O’Day EM, Waudby CA, Meehan S, Dumoulin M, Hsu S-TD, Cremades N, Verschueren KHG, et al. Structure and properties of a complex of α-synuclein and a single-domain camelid antibody. *J Mol Biol* (2010) **402**:326–43. doi:10.1016/j.jmb.2010.07.001

31. Shahangian SS, H. Sajedi R, Hasannia S, Jalili S, Mohammadi M, Taghdir M, Shali A, Mansouri K, Sariri R. A conformation-based phage-display panning to screen neutralizing anti-VEGF VHHs with VEGFR2 mimicry behavior. *Int J Biol Macromol* (2015) **77**:222–234. doi:10.1016/j.ijbiomac.2015.02.047

32. Jittavisutthikul S, Thanongsaksrikul J, Thueng-in K, Chulanetra M, Srimanote P, Seesuay W, Malik A, Chaicumpa W. Humanized-VHH Transbodies that Inhibit HCV Protease and Replication. *Viruses* (2015) **7**:2030–2056. doi:10.3390/v7042030

33. Unger M, Eichhoff AM, Schumacher L, Strysio M, Menzel S, Schwan C, Alzogaray V, Zylberman V, Seman M, Brandner J, et al. Selection of Nanobodies that Block the Enzymatic and Cytotoxic Activities of the Binary Clostridium Difficile Toxin CDT. *Sci Rep* (2015) **5**:7850. doi:10.1038/srep07850

34. Fridy PC, Thompson MK, Ketaren NE, Rout MP. Engineered high-affinity nanobodies recognizing staphylococcal Protein A and suitable for native isolation of protein complexes. *Anal Biochem* (2015) **477**:92–94. doi:10.1016/j.ab.2015.02.013

35. Xiong X, Martin SR, Haire LF, Wharton SA, Daniels RS, Bennett MS, McCauley JW, Collins PJ, Walker PA, Skehel JJ, et al. Receptor binding by an H7N9 influenza virus from humans. *Nature* (2013) **499**:496–9. doi:10.1038/nature12372

36. Dolk E, van der Vaart M, Lutje Hulsik D, Vriend G, de Haard H, Spinelli S, Cambillau C, Frenken L, Verrips T. Isolation of llama antibody fragments for prevention of dandruff by phage display in shampoo. *Appl Environ Microbiol* (2005) **71**:442–50. doi:10.1128/AEM.71.1.442-450.2005

37. Calpe S, Wagner K, El Khattabi M, Rutten L, Zimberlin C, Dolk E, Verrips CT, Medema JP, Spits H, Krishnadath KK. Effective Inhibition of Bone Morphogenetic Protein Function by Highly Specific Llama-Derived Antibodies. *Mol Cancer Ther* (2015) **14**:

38. Schmitz KR, Bagchi A, Roovers RC, van Bergen en Henegouwen PMP, Ferguson KM. Structural Evaluation of EGFR Inhibition Mechanisms for Nanobodies/VHH Domains. *Structure* (2013) **21**:1214–1224. doi:10.1016/j.str.2013.05.008

39. Park Y-J, Budiarto T, Wu M, Pardon E, Steyaert J, Hol WGJ. The structure of the C-terminal domain of the largest editosome interaction protein and its role in promoting RNA binding by RNA-editing ligase L2. *Nucleic Acids Res* (2012) **40**:6966–77. doi:10.1093/nar/gks369

40. Thompson JD, Higgins DG, Gibson TJ. CLUSTAL W: improving the sensitivity of progressive multiple sequence alignment through sequence weighting, position-specific gap penalties and weight matrix choice. *Nucleic Acids Res* (1994) **22**:4673–80.

41. Laskowski RA, MacArthur MW, Moss DS, Thornton JM, IUCr. PROCHECK: a program to check the stereochemical quality of protein structures. *J Appl Crystallogr* (1993) **26**:283–291. doi:10.1107/S0021889892009944

42. Prado NDR, Pereira SS, da Silva MP, Morais MSS, Kayano AM, Moreira-Dill LS, Luiz MB, Zanchi FB, Fuly AL, E. F. Huacca M, et al. Inhibition of the Myotoxicity Induced by Bothrops jararacussu Venom and Isolated Phospholipases A2 by Specific Camelid Single-Domain Antibody Fragments. *PLoS One* (2016) **11**:e0151363. doi:10.1371/journal.pone.0151363

43. Jullien D, Vignard J, Fedor Y, Béry N, Olichon A, Crozatier M, Erard M, Cassard H, Ducommun B, Salles B, et al. Chromatibody, a novel non-invasive molecular tool to explore and manipulate chromatin in living cells. *J Cell Sci* (2016)jcs.183103. doi:10.1242/jcs.183103

44. Parks WC, Mecham RP. *Matrix metalloproteinases*. Academic Press (1998).

45. Acharya P, Luongo TS, Georgiev IS, Matz J, Schmidt SD, Louder MK, Kessler P, Yang Y, McKee K, O’Dell S, et al. Heavy chain-only IgG2b llama antibody effects near-pan HIV-1 neutralization by recognizing a CD4-induced epitope that includes elements of coreceptor- and CD4-binding sites. *J Virol* (2013) **87**:10173–81. doi:10.1128/JVI.01332-13

46. Korotkov K V, Pardon E, Steyaert J, Hol WGJ. Crystal structure of the N-terminal domain of the secretin GspD from ETEC determined with the assistance of a nanobody. *Structure* (2009) **17**:255–65. doi:10.1016/j.str.2008.11.011

47. Morgado I, Wieligmann K, Bereza M, Rönicke R, Meinhardt K, Annamalai K, Baumann M, Wacker J, Hortschansky P, Malešević M, et al. Molecular basis of β-amyloid oligomer recognition with a conformational antibody fragment. *Proc Natl Acad Sci U S A* (2012) **109**:12503–8. doi:10.1073/pnas.1206433109

48. Pain C, Kerff F, Herman R, Sauvage E, Preumont S, Charlier P, Dumoulin M, Pardon E, Matagne A, Charlier P, et al. Structure of a single-domain camelid antibody fragment cAb-H7S specific of the BlaP beta-lactamase from Bacillus licheniformis. *doi.org* doi:10.2210/pdb4m3j/pdb

49. Demeestere D, Dejonckheere E, Steeland S, Hulpiau P, Haustraete J, Devoogdt N, Wichert R, Becker-Pauly C, Van Wonterghem E, Dewaele S, et al. Development and Validation of a Small Single-domain Antibody That Effectively Inhibits Matrix Metalloproteinase 8. *Mol Ther* (2016) **24**:890–902. doi:10.1038/mt.2016.2

50. Bioinformatics B, Chen C-C, Hwang J-K, Yang J-M. (PS) 2 -v2: template-based protein structure prediction server. doi:10.1186/1471-2105-10-366

51. Sippl MJ. Recognition of errors in three-dimensional structures of proteins. *Proteins Struct Funct Genet* (1993) **17**:355–362. doi:10.1002/prot.340170404

52. Wiederstein M, Sippl MJ. ProSA-web: interactive web service for the recognition of errors in three-dimensional structures of proteins. *Nucleic Acids Res* (2007) **35**:W407-10. doi:10.1093/nar/gkm290

53. Sefid F, Rasooli I, Payandeh Z. Homology modeling of a Camelid antibody fragment against a conserved region of Acinetobacter baumannii biofilm associated protein (Bap). *J Theor Biol* (2016) **397**:43–51. doi:10.1016/j.jtbi.2016.02.015

54. Dunbar J, Krawczyk K, Leem J, Baker T, Fuchs A, Georges G, Shi J, Deane CM. SAbDab: the structural antibody database. *Nucleic Acids Res* (2014) **42**:D1140-6. doi:10.1093/nar/gkt1043

55. Leem J, Dunbar J, Georges G, Shi J, Deane CM. ABodyBuilder: Automated antibody structure prediction with data–driven accuracy estimation. *MAbs* (2016) **8**:1259–1268. doi:10.1080/19420862.2016.1205773

56. Vincke C, Loris R, Saerens D, Martinez-Rodriguez S, Muyldermans S, Conrath K. General strategy to humanize a camelid single-domain antibody and identification of a universal humanized nanobody scaffold. *J Biol Chem* (2009) **284**:3273–84. doi:10.1074/jbc.M806889200

57. Saerens D, Pellis M, Loris R, Pardon E, Dumoulin M, Matagne A, Wyns L, Muyldermans S, Conrath K. Identification of a universal VHH framework to graft non-canonical antigen-binding loops of camel single-domain antibodies. *J Mol Biol* (2005) **352**:597–607. doi:10.1016/j.jmb.2005.07.038

58. Soler MA, de Marco A, Fortuna S, Wall R, Wan Y. Molecular dynamics simulations and docking enable to explore the biophysical factors controlling the yields of engineered nanobodies. *Sci Rep* (2016) **6**:34869. doi:10.1038/srep34869

59. Desmyter A, Farenc C, Mahony J, Spinelli S, Bebeacua C, Blangy S, Veesler D, van Sinderen D, Cambillau C. Viral infection modulation and neutralization by camelid nanobodies. *Proc Natl Acad Sci U S A* (2013) **110**:E1371-9. doi:10.1073/pnas.1301336110

60. Peng J, Xu J. RaptorX: exploiting structure information for protein alignment by statistical inference. *Proteins* (2011) **79 Suppl 10**:161–71. doi:10.1002/prot.23175

61. Källberg M, Wang H, Wang S, Peng J, Wang Z, Lu H, Xu J. Template-based protein structure modeling using the RaptorX web server. *Nat Protoc* (2012) **7**:1511–1522. doi:10.1038/nprot.2012.085

62. Ko J, Lee D, Park H, Coutsias EA, Lee J, Seok C. The FALC-Loop web server for protein loop modeling. *Nucleic Acids Res* (2011) **39**:W210–W214. doi:10.1093/nar/gkr352

63. Malik A, Imtong C, Sookrung N, Katzenmeier G, Chaicumpa W, Angsuthanasombat C. Structural Characterization of Humanized Nanobodies with Neutralizing Activity against the Bordetella pertussis CyaA-Hemolysin: Implications for a Potential Epitope of Toxin-Protective Antigen. *Toxins (Basel)* (2016) **8**:99. doi:10.3390/toxins8040099
