# Supplementary material for: Discrete analysis of camelid variable domains: sequences, structures, and in-silico structure prediction
Source: PeerJ. 2020 Mar 6;8:e8408. doi: 10.7717/peerj.8408 (PMC7061911; doi:10.7717/peerj.8408)
Supplement: Supplemental Information 2 [file peerj-08-8408-s023.docx]

Though it is understandable that one observes PB variability in CDRs, there is conformational diversity also in non-CDRs such as 7-17, 41-45, 65-68 and 74-80. The latter region belongs to the much-debated “CDR4”, in the former regions, most of the residues in the first region and all residues in the second and third regions include the loops towards the C-terminal end. Further, the *N_eq_* values of case where template temp-m is used is mostly low compared to the multi-template case as well as the other cases modeled using single templates, expected in the amino acid region of 65-68. The models from multiple templates show maximum *N_eq_* in the CDR2 region. The residue composition of CDR2 is in the query is ‘SW***SGG***STY’, in which the three residues italicized are the positions with high *N_eq_* values. As the two glycines do not provide lot of constraints to Modeller software due to the poor sequence identity with templates, it allowed a large conformational space search. The major PB signature of this region being *fklopac*, a classical succession of PBs observed mainly in α to β loop [77].
