# Supplementary material for: Discrete analysis of camelid variable domains: sequences, structures, and in-silico structure prediction
Source: PeerJ. 2020 Mar 6;8:e8408. doi: 10.7717/peerj.8408 (PMC7061911; doi:10.7717/peerj.8408)
Supplement: Supplemental Information 3 [file peerj-08-8408-s024.docx]

We carefully dissected various parameters like the sequence identity and structural similarity (local and global), to be able to assess conformations of the models generated better. We explored the conformational changes associated with the generated models in each case using Protein Blocks. The outcome of our analysis shows that the model conformational diversity not only in the expected CDRs but also in the FRs. Additionally, using second best template (in terms of sequence identity) temp-m is the best possible scenario for the query structure prediction compared to other templates. We also report that the number of residue positions which have *N_eq_* > 1 are the most in case of multi-template modeling, which are due to the influence of multiple templates at that position. The second-best template in terms of sequence identity (temp-m) is the best template for modeling the query amongst all the scenarios as it has the least number of residues positions with *N_eq_* > 1, whereas the first best template in terms of sequence identity (temp-l) is the third best in terms of residues with *N_eq_* >1. A PB profile comparison of values between generated models and by multiple templates are than in other cases, suggesting that temp-m has influenced the model conformations in the later scenario.
